# Damage response protein 1 (Dap1) functions in the synthesis of carotenoids and sterols in *Xanthophyllomyces dendrorhous*

**DOI:** 10.1016/j.jlr.2022.100175

**Published:** 2022-02-02

**Authors:** Ana-María González, Maximiliano Venegas, Salvador Barahona, Melissa Gómez, María-Soledad Gutiérrez, Dionisia Sepúlveda, Marcelo Baeza, Víctor Cifuentes, Jennifer Alcaíno

**Affiliations:** 1 Departamento de Ciencias Ecológicas, Facultad de Ciencias, Universidad de Chile, Santiago, Chile; 2 Centro de Biotecnología, Facultad de Ciencias, Universidad de Chile, Santiago, Chile

**Keywords:** cytochrome P450, isoprenoids, nuclear receptors/SREBP, sterols, astaxanthin, *X. dendrorhous*, Dap1, Cyp51, Cyp61, CrtS, CBR, CYB5 reductase, Co-IP, coimmunoprecipitation, CPR, cytochrome P450 reductase, CrtS, astaxanthin synthase, CYB5, cytochrome b5, Dap1, damage response protein 1, P450, cytochrome P450, PGRMC1, progesterone membrane receptor component 1, qPCR, quantitative PCR, RP-HPLC, reverse-phase HPLC, YEP, yeast extract peptone, YM, yeast malt

## Abstract

Cytochrome P450s (P450s) are heme-containing proteins involved in several cellular functions, including biosynthesis of steroidal hormones, detoxification of xenobiotic compounds, among others. Damage response protein 1 (Dap1) has been described as a positive regulator of P450s through protein-protein interactions in organisms such as *Schizosaccharomyces pombe*. Three P450s in the carotenogenic yeast *Xanthophyllomyces dendrorhous* have thus far been characterized: Cyp51 and Cyp61, which are involved in ergosterol biosynthesis, and CrtS (astaxanthin synthase), which is involved in biosynthesis of the carotenoid astaxanthin. In this work, we describe the *X. dendrorhous DAP1* gene, deletion of which affected yeast pigmentation by decreasing the astaxanthin fraction and increasing the β-carotene (a substrate of CrtS) fraction, which is consistent with the known role of CrtS. We found that the proportion of ergosterol was also decreased in the *Δdap1* mutant. However, even though the fractions of the end products of these two pathways (the synthesis of carotenoids and sterols) were decreased in the *Δdap1* mutant, the transcript levels of genes from the P450 systems involved were higher than those in the wild-type strain. We demonstrate that Dap1 coimmunoprecipitates with these three P450s, suggesting that Dap1 interacts with these three proteins. We propose that Dap1 regulates the synthesis of astaxanthin and ergosterol in *X. dendrorhous*, probably by regulating the P450s involved in both biosynthetic pathways at the protein level. This work suggests a new role for Dap1 in the regulation of carotenoid biosynthesis in *X. dendrorhous*.

Cytochrome P450 enzymes (P450s or CYPs) belong to a large superfamily of heme-containing proteins responsible for catalyzing the oxidation of a wide variety of both endogenous and exogenous compounds (1). These enzymes are involved in the oxidative metabolism of steroids, fatty acids, prostaglandins, and pheromone plant metabolites, among others (2). P450s carry out their function by inserting an oxygen atom from molecular oxygen (O_2_) into an organic substrate (RH), whereas the second oxygen from molecular oxygen is reduced to water by the consumption of two reducing equivalents to form ROH, as in the following general reaction: RH + O_2_ + 2e^−^ + 2H^+^ → ROH + H_2_O (3). The required electrons are generally supplied by NADPH and transferred to P450 by a P450 redox partner, forming the P450 system. In class II eukaryotic microsomal P450 systems (the most common class in eukaryotic organisms), the general P450 redox partner is a cytochrome P450 reductase (CPR), the dual flavoprotein CPR (4).

Another protein that interacts with P450s and transfers electrons to them at the endoplasmic reticulum is the hemoprotein cytochrome b5 (CYB5). In this case, CYB5 may receive electrons from NADH from a CYB5 reductase (CBR) (5), constituting an alternative electron donor system. In the past, CPR and CYB5 were the only known proteins capable of functionally interacting with P450s to carry out their monooxygenase activity at the endoplasmic reticulum membrane (6). Recently, new candidate proteins have been described that interact with P450s; among these candidate proteins is a member of the membrane-associated progesterone receptor family: the progesterone membrane receptor component 1 (PGRMC1)/damage response protein 1 (Dap1). PGRMC1 in humans has been characterized as a small protein of approximately 22 kDa with a transmembrane segment at its N-terminus and a CYB5-like domain at its C-terminus (7). In mammals, PGRMC1 is required for CYP51A1 activity (protein involved in the demethylation of lanosterol in the synthesis of cholesterol) (8). In addition, other P450s may also require PGRMC1 for their activity, for example, CYP3A4, which strongly binds with PGRMC1 (8). Studies carried out in mice have shown that PGRMC1 is capable of binding to more than 13 P450s, helping them to maintain their protein levels (9).

The homologous protein in *Saccharomyces cerevisiae*, Dap1, is involved in the response to DNA damage and has been studied in the context of the identification of new genes (10). The deletion of *DAP1* in this yeast increased azole sensitivity, reduced ergosterol (the main sterol in yeasts) production, and increased the proportion of intermediate sterols, such as lanosterol. Lanosterol is a substrate of the P450 Erg11/Cyp51 (lanosterol-14-demethylase), so this result suggests a partial defect in the activity of this enzyme (11). In addition, a model in which heme binding by Dap1 is required to activate Erg11/Cyp51 was proposed, although the Dap1-Erg11 complex could not be directly detected by coimmunoprecipitation (co-IP) experiments (11). In the fission yeast *Schizosaccharomyces pombe*, *DAP1* mutation also altered sterol production, and protein-protein interactions between Dap1 and two P450s from this microorganism, Erg11/Cyp51 and Erg5/Cyp61 (C-22 sterol desaturase), and between Dap1 and P450s from other organisms involved in different cellular processes were detected; Dap1 was thus proposed to be a general modulator of P450s, as it could interact with several P450s (8).

The basidiomycete yeast *Xanthophyllomyces dendrorhous* produces astaxanthin, a carotenoid with biotechnological potential mainly for its use in aquaculture for salmonid pigmentation (12). Thirteen potential P450-encoding genes have been identified and bioinformatically characterized in this yeast (13), and among these genes, three have been functionally characterized; two are involved in ergosterol biosynthesis (Erg11/Cyp51 (14) and Erg5/Cyp61 (15)), and one is involved in the biosynthesis of CrtS (astaxanthin synthase) (16). CrtS catalyzes the last steps of astaxanthin production from β-carotene and exclusively uses the CPR, named as CrtR in this organism (encoded by the *crtR* gene) (17), as an electron donor (Fig. 1). Ergosterol was still produced in *crtR*^*−*^ mutant *X. dendrorhous*, suggesting that the P450s involved in this process can use the alternative electron donor system formed by CYB5-CBR (18). Until now, no other proteins involved in the activity of P450s, such as Dap1, had been identified and characterized in this microorganism. Considering the biological relevance of P450 systems and the importance of the P450 CrtS in the biosynthesis of astaxanthin in *X. dendrorhous*, in this work, the yeast *DAP1* gene was identified and functionally characterized. In addition, protein-protein interactions between Dap1 and the P450s Cyp51, Cyp61, and CrtS were confirmed by co-IP.

**Fig. 1:**
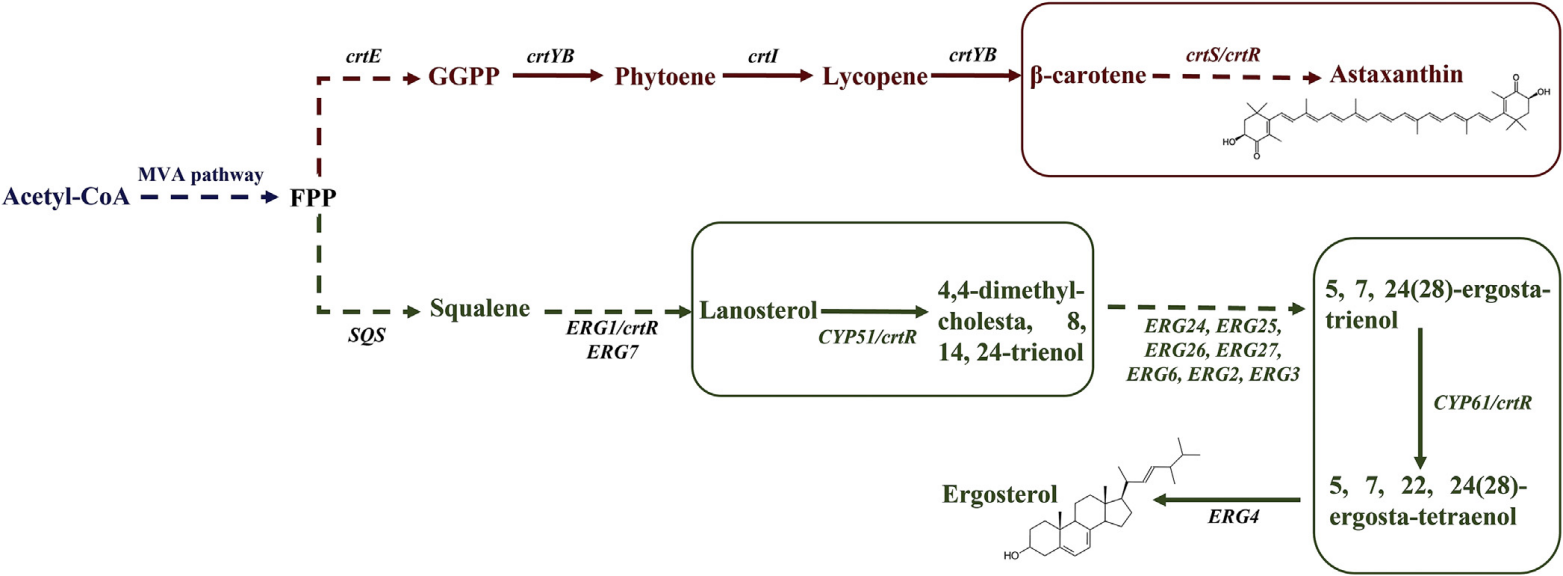
Biosynthesis of astaxanthin and ergosterol. The production of carotenoids and sterols derives from the mevalonate (MVA) pathway. Astaxanthin biosynthesis begins with geranylgeranyl pyrophosphate (GGPP) and ergosterol biosynthesis with squalene, both deriving from farnesyl pyrophosphate (FPP). Squares enclose the reactions involving P450 enzymes (astaxanthin synthase [CrtS], lanosterol-14-demethylase, and C-22 sterol desaturase, encoded by the *crtS*, *CYP51*, and *CYP61* genes, respectively).

## Materials and methods

### Strains, plasmids, and growth conditions

All strains used and constructed in this work are listed in Table 1. All *X. dendrorhous* strains were derived from the wild-type strain CBS 6938 (ATCC 96594). The CBSTr strain (*crtR*^*−*^ mutant) (17) was also used in phenotypic analysis for comparative purposes. For most experiments, *X. dendrorhous* strains were cultured in yeast malt (YM) medium (0.3% yeast extract, 0.3% malt extract, 0.5% peptone, and 1% glucose) with constant agitation at 22°C. For the selection of transformant colonies, YM agar (1.5%) medium was supplemented with hygromycin B (35 μg/ml) and/or zeocin (45 μg/ml) according to the genotype of the strain. Growth curves were constructed using triplicate cultures registering growth by measuring the absorbance of the cultures at 600 nm using a V-630 UV-Vis spectrophotometer from JASCO (JASCO, Easton, MD). To construct the plasmids that were used to generate strains CBS.*DAP1.FLAG*, CBS.*DAP1.FLAG-CYP61.HA*, CBS.*DAP1.FLAG-CYP51.HA*, and CBS.*DAP1.FLAG-crtS.HA* (supplemental Fig. S1), DNA assembler methodology was used in *S. cerevisiae* (23, 24), in which PCR-amplified DNA fragments were assembled in vivo through homologous recombination. *S. cerevisiae* strains were cultured at 30°C in yeast extract peptone (YEP) medium (2% glucose, 1% yeast extract, and 2% peptone). For transformant selection, YEP agar medium was supplemented with the antibiotic G-418 (200 μg/ml). *E. coli* was cultured at 37°C with constant agitation in lysogeny broth medium (1% tryptone, 0.5% yeast extract, and 0.5% NaCl) and on 1.5% agar lysogeny broth plates supplemented with 100 μg/ml ampicillin for plasmid selection and 20 μg/ml X-gal (5-bromo-4-chloro-3-indolyl-β-d-galactopyranoside) for recombinant clone selection by blue-white screening.

**Table 1:** Strains and plasmids used and/or constructed in this work

Strains/Plasmid	Description	Reference or Source
Strain		
*E. coli*		
DH5α	Amp^S^. Used for molecular cloning and plasmid maintenance	(19)
*S. cerevisiae*		
S288c	Haploid reference strain used for plasmid construction by DNA assembler	ATCC 204508
*X. dendrorhous*		
CBS 6938	Wild-type strain (Zeo^S^ and Hyg^S^)	ATCC 96594
CBSTr	Mutant (Zeo^S^ and Hyg^R^) derived from CBS 6938. The single *crtR* locus was interrupted with the hygromycin B resistance cassette	(17)
CBS.*Δdap1*	Mutant (Zeo^S^ and Hyg^R^) derived from CBS 6938. The single *DAP1* locus was replaced with the hygromycin B resistance cassette	This work
CBS.*sre1*^*−*^	Mutant (Zeo^R^ and Hyg^S^) derived from CBS 6938. Gene *SRE1* was partially deleted (approximately 90% of the coding region was replaced with the zeocin resistance cassette)	(20)
CBS.*sre1*^*−*^.*Δdap1*	Mutant (Zeo^R^ and Hyg^R^) derived from CBS.*sre1*^*−*^. The single *DAP1* locus was replaced with the hygromycin B resistance cassette	This work
CBS.*DAP1.FLAG*	Mutant (Zeo^S^ and Hyg^R^) derived from CBS 6938. The native *DAP1* gene was replaced with a gene variant that expresses the Dap1 protein fused to the 3xFLAG epitope at its C-terminus, followed by the hygromycin B resistance cassette	This work
CBS.*DAP1.FLAG-CYP61.HA*	Mutant (Zeo^S^ and Hyg^R^) derived from CBS.*DAP1.FLAG*. The native *CYP61* gene was replaced with a gene variant that expresses the Cyp61 protein fused to the 3xHA epitope at its C-terminus, followed by the zeocin resistance cassette	This work
CBS.*DAP1.FLAG-CYP51.HA*	Mutant (Zeo^S^ and Hyg^R^) derived from CBS.*DAP1.FLAG*. The native *CYP51* gene was replaced with a gene variant that expresses the Cyp51 protein fused to the 3xHA epitope at its C-terminus, followed by the zeocin resistance cassette	This work
CBS.*DAP1.FLAG-crtS.HA*	Mutant (Zeo^S^ and Hyg^R^) derived from CBS.*DAP1.FLAG*. The native *crtS* gene was replaced with a gene variant that expresses the CrtS protein fused to the 3xHA epitope at its C-terminus, followed by the zeocin resistance cassette	This work
Plasmid		
pBlueScript SK-(pBS)	Cloning vector (ColE1 ori, Amp^R^, blue-white colony selection)	Agilent Technologies, Inc, Santa Clara, CA
pMN-*hph*	pBS containing the hygromycin B resistance cassette (1.8 kb) used for *X. dendrorhous* transformant selection at the EcoRV site	(21)
pIR-*zeo*	pBS containing the zeocin resistance cassette (1.2 kb) used for *X. dendrorhous* transformant selection at the EcoRV site	(15)
pBS-Δg*DAP1*-*hph*	pBS containing the 662 bp upstream and 642 bp downstream of the *DAP1* gene and the hygromycin B resistance module between them at the EcoRV site. Used to delete the *X. dendrorhous DAP1* gene by homologous recombination	This work
pXd-*DAP1.FLAG-hyg*	Plasmid constructed by DNA assembler used to replace the *X. dendrorhous DAP1* gene with the gene variant that expresses Dap1 3xFLAG-tagged at its C-terminal end. It contains 935 bp of the genomic *DAP1* gene (including 130 bp upstream the translation start codon followed by 805 bp of the gene without the stop codon) fused to the 3xFLAG-encoding sequence at the 3′ end. This sequence is followed by the downstream region of the *DAP1* gene to direct its integration at the *DAP1* locus, including a hygromycin B resistance cassette for transformant selection	This work
pXD-*CYP61.HA*-*zeo*	Plasmid constructed by DNA assembler used to replace the *X. dendrorhous CYP61* gene with the gene variant that expresses Cyp61 3xHA-tagged at its C-terminal end. It contains 2,680 bp of the genomic *CYP61* gene (including 140 bp upstream the translation start codon followed by 2,540 bp of the gene without the stop codon) fused to the 3xHA-encoding sequence at the 3′ end. This sequence is followed by the downstream region of the *CYP61* gene to direct its integration at the *CYP61* locus including a zeocin resistance cassette for transformant selection	This work
pXD-*CYP51.HA*-*zeo*	Plasmid constructed by DNA assembler used to replace the *X. dendrorhous CYP51* gene with the gene variant that expresses Cyp51 3xHA-tagged at its C-terminal end. It contains 2,812 bp of the genomic *CYP51* gene (including 89 bp upstream the translation start codon followed by 2,723 of the gene without the stop codon) fused to the 3xHA-encoding sequence at the 3′ end. This sequence is followed by the downstream region of the *CYP51* gene to direct its integration at the *CYP51* locus, including a zeocin resistance cassette for transformant selection	This work
pXD-*crtS.HA*-*zeo*	Plasmid constructed by DNA assembler used to replace the *X. dendrorhous crtS* gene with the gene variant that expresses CrtS 3xHA-tagged at its C-terminal end. It contains 3,394 bp of the genomic *crtS* gene (including 231 bp upstream the translation start codon followed by 3,163 bp of the gene without the stop codon) fused to the 3xHA-encoding sequence at the 3′ end. This sequence is followed by the downstream region of the *crtS* gene to direct its integration at the *crtS* locus, including a zeocin resistance cassette for transformant selection	This work
pYES2	*S. cerevisiae* expression vector containing the 2μ origin. Used to amplify the 2μ DNA by PCR, which was then used for plasmid construction by DNA assembler	Thermo Fisher Scientific, Inc, Waltham, MA
pFA6	Yeast plasmid with kanamycin/geneticin (G418) resistance marker. Used to amplify the G418 marker by PCR, which was then used for plasmid construction by DNA assembler	(22)

Amp^S^/Amp^R^, sensitive/resistant to ampicillin; ColE1 ori, replication origin of *E. coli* ColE1 plasmid; Hyg^S^/Hyg^R^, sensitive/resistant to hygromycin B; Zeo^S^/Zeo^R^, sensitive/resistant to zeocin.

For phenotypic assays using the microdrop technique, YM agar medium supplemented with the azoles clotrimazole, ketoconazole, or itraconazole was used. For these assays, cultures of the strains under study were serially 10-fold diluted, and a 5 μl drop of each dilution was deposited on the plate.

### Bioinformatic analyses

The *DAP1* gene of *X. dendrorhous* was identified by local BLASTp search of genomic and transcriptomic data from the *X. dendrorhous* strain UCD 67-385 (ATCC 24230) (25) with Geneious R11 using related sequences obtained from the GenBank database as queries. Protein sequence analyses were performed with programs available at http://www.ebi.ac.uk/interpro (26) and https://embnet.vital-it.ch/software/TMPRED_form.html (27).

### Nucleic acid extraction, DNA amplification, and sequence analysis

Yeast genomic DNA extraction was performed by mechanical rupture with glass beads (19). RNA extraction was carried out from the cell pellet of a stationary phase culture (2 ml) that had been suspended in 200 μl of lysis buffer (2 mM sodium acetate, pH 5.5%, 0.5% SDS, and 1 mM EDTA), to which 100 μl of 0.5 mm glass beads were added. Each sample was homogenized by vortex agitation for 5 min at 4°C, and then 800 μl of TRI reagent (Life Technologies, Carlsbad, CA) was added. Each sample was homogenized again by vortex agitation for 5 min at 4°C and then incubated at room temperature for 10 min. Next, 200 μl of chloroform was added, and the mixture was manually stirred for 15 s and incubated at room temperature for 6 min. Samples were centrifuged at 18,440 *g* for 15 min at 4°C, and the aqueous phase was recovered. The aqueous phase was deposited in two microcentrifuge tubes, and 250 μl of sterile water and 550 μl of cold isopropanol were added to each tube, which was then incubated at room temperature for 10 min. Then, the tube was centrifuged for 15 min at 18,440 *g* at 4°C, the supernatant was removed, and the pellet was washed with 1 ml of 70% ethanol and centrifuged again for 5 min. Finally, the pellet was suspended in 20–30 μl of sterile water. Total RNA was quantified spectrophotometrically at 260 nm. Plasmid DNA was obtained from *E. coli* using the Gene JET Plasmid Miniprep Kit (Thermo Fisher Scientific, Waltham, MA).

All oligonucleotides used in this study were purchased from Integrated DNA Technologies (Coralville, IA) and are listed in supplemental Table S1. PCR amplification for the analysis of plasmids and clones generated in this work was performed using *Taq* DNA polymerase. DNA fragments that were subsequently assembled by DNA assembler methodology were amplified with *Pfu* DNA polymerase. In both cases, PCR was performed in a final volume of 25 μl containing 1× PCR buffer (200 mM Tris-HCl [pH 8.4], 500 mM KCl), 2 mM MgCl_2_, 1× BCP (spanish acronym for PCR loading buffer) loading buffer (100 mM cresol red, 8.3% glycerol), 200 μM each deoxynucleotide (deoxynucleoside triphosphate), 1 μM each primer, 1 U of DNA polymerase enzyme (*Taq* or *Pfu*), and 10–20 ng of template DNA. PCR was performed in a 2720 thermal cycler (Applied Biosystems, Foster City, CA) using the following general program: initial denaturation at 94°C for 3 min and 35 cycles of denaturation at 94°C for 30 s, primer hybridization at 55°C for 30 s, and elongation at 72°C for 3–4 min (depending on the size of the amplified product). After 35 cycles, a final elongation step at 72°C was carried out, and the reaction was kept at 4°C until analysis. DNA sequencing was performed via services from Macrogen, Inc (Seoul, Korea), and sequences were analyzed mainly with Geneious R11 (28) software (https://www.geneious.com by Biomatters) and programs available online at the NCBI website.

### Single-strand DNA synthesis and quantitative PCR (RT-qPCR)

Complementary DNA synthesis was performed with M-MLV reverse transcriptase (Thermo Fisher Scientific) according to the manufacturer’s protocol with 5 μg of total RNA in a final volume of 20 μl using oligo-dT18. Relative transcript level determination was performed in an Mx3000P qPCR system (Stratagene, San Diego, CA) using primer pairs with efficiencies greater than 95%, as determined by standard curves. Each reaction contained 1 μl of the RT reaction, each primer at 0.25 μM and 10 μl of the reagent SensiMix SYBR Green I (Quantace, London, GBR) at a final volume of 20 μl. The obtained cycle threshold (Ct) values were normalized to the corresponding value for *X. dendrorhous* beta-actin (GenBank: X89898.1) and later expressed as a function of control conditions (wild-type strain) using the ΔΔCt algorithm (29).

### Plasmid construction

All plasmids used in this work are listed in Table 1. The *DAP1* gene was knocked out in *X. dendrorhous* using the plasmid pBS-Δg*DAP1-hph*. This plasmid was constructed by joining the 662-bp upstream and 642-bp downstream DNA regions of the gene, which had been PCR amplified from genomic DNA. DNA fragments were joined by overlap extension PCR (30), leaving an HpaI restriction site between them that was included because of the design of the primers, and then ligated at the EcoRV site of the pBluescript SK-plasmid. Finally, pBS-Δg*DAP1-hph* was generated by inserting an antibiotic resistance cassette (hygromycin B) at the HpaI site for *X. dendrorhous* transformant selection. For plasmid construction by DNA assembler, the DNA fragments to be assembled were designed such that they shared 80 bp of identical sequence at their ends to allow recombination.

### Yeast transformation

Electrocompetent cells were prepared from a saturated culture of *S. cerevisiae* grown in YEP medium at 30°C at an absorbance of 1.3–1.5 at 600 nm. Cells were collected by centrifugation and washed twice with a one-half volume of sterile cold water. Subsequently, the cells were washed with a one-quarter volume of cold 1 M sorbitol, suspended in 1–2 ml of 1 M sorbitol, and separated into 100 μl fractions. For the DNA assembler method, an aliquot of electrocompetent cells was mixed with 2 μl of each PCR fragment (previously dialyzed) to be assembled and transferred to a 2 mm electroporation cuvette. A pulse of 1,500 V, 25 μF, and 200 Ω was applied using a Gene Pulser Xcell electroporator (BioRad Laboratories, Inc, Hercules, CA). *X. dendrorhous* transformation was also performed by electroporation as previously described (31, 32) using 1–5 μg of linear transformant DNA. Electrocompetent cells were prepared from exponential cultures, grown in YM medium, and electroporated using a Gene Pulser Xcell electroporator with Pulse Controller and Capacitance Extender modules (BioRad Laboratories, Inc, Hercules, CA) under the following conditions: 125 mF, 600 Ω, and 0.45 kV.

### Sterol and carotenoid extraction and analysis

Carotenoids (33) and sterols (34) were extracted according to previous methods, quantified spectrophotometrically, and normalized to the dry weight of the yeast. Carotenoids were quantified at 465 nm using an absorption coefficient of A_1%_ = 2,100, and sterols were quantified at 280 nm using an absorption coefficient of A_1%_ = 11,500. The extracted carotenoids and sterols were separated by reverse-phase HPLC (RP-HPLC) using an RP-18 Lichrocart 125-4 column (Merck KGaA, Darmstadt, Germany) with acetonitrile:methanol:isopropanol (85:10:5, v/v/v) and methanol:water (97:3, v/v) as the mobile phase, respectively, with a 1 ml/min flux under isocratic conditions. The elution spectra were recovered using a diode array detector; carotenoids and sterols were identified according to comparison of their spectra and retention time to those of standards.

### Protein extraction and co-IP

*X. dendrorhous* strains CBS 6938, CBS.*DAP1.FLAG*, CBS.*DAP1.FLAG-CYP51.HA*, and CBS.*DAP1.FLAG-crtS.HA* were grown in YM medium at 22°C with constant agitation for 72 h, and the cell pellet from 3 ml of culture was suspended in 250 μl of lysis buffer (100 mM NaHCO_3_, 0.5% Triton X-100, 1 mM PMSF, 1× protease inhibitor (Promega, Madison, WI), and 2 mM Tris(2-carboxyethyl)phosphine), and 0.1 ml glass beads (0.5 mm). In the case of strain CBS.*DAP1.FLAG-CYP61.HA*, the cell pellet from 9 ml of culture was collected. In all cases, seven cycles of mechanical cell disruption for 30 s were performed using a Mini-BeadBeater-16 (BioSpec Products, Inc, Bartlesville, OK), with the samples incubated on ice for 1 min between each cycle. Subsequently, centrifugation was performed at 4°C for 10 min at 18,440 *g*, after which the supernatant was recovered in a clean tube. After extraction by means of Millex-HV filters (Merck Millipore, Burlington, MA), the filtered samples were quantified using a Pierce BCA protein test kit (Thermo Fisher Scientific), and their concentrations were equalized according to the least concentrated sample. However, in the case of strain CBS.*FLAG.DAP1*-*CYP61.HA*, the obtained protein samples were 4-fold more concentrated than protein samples from the other strains. The Pierce™ Classic Magnetic IP/Co-IP kit (Thermo Fisher Scientific) with MagnaBind™ Magnet (Thermo Fisher Scientific) was used following the manufacturer's instructions to carry out co-IP. Protein transfer from SDS-PAGE gels was performed under semidry conditions at 15 V for 30 min using a Trans-Blot® SD Transfer Cell (Bio-Rad Laboratories, Inc, Hercules, CA). For Western blot analysis, the monoclonal antibody anti-FLAG® M2 (catalog number: F1804; Sigma-Aldrich, Saint Louis, MO) at a dilution of 1:1,000 or 50 mU of high-affinity anti-HA peroxidase (Roche, Basel, CHE) was used. Monoclonal antiubiquitin antibody (catalog number: SAB2702288; Sigma-Aldrich) at a dilution of 1:1,000 was used as a loading control. In the case of Western blotting with anti-FLAG and antiubiquitin, anti-mouse IgG H&L (whole molecule)-peroxidase antibody (catalog number: A9044; Sigma-Aldrich) at a dilution of 1:5,000 was used as a secondary antibody.

### Statistical analysis

Statistically significant differences between strains or experimental conditions were identified using one-way ANOVA and Tukey post hoc tests.

## Results

### Isolation and sequence analysis of the *X. dendrorhous DAP1* gene

**Fig. 2:**
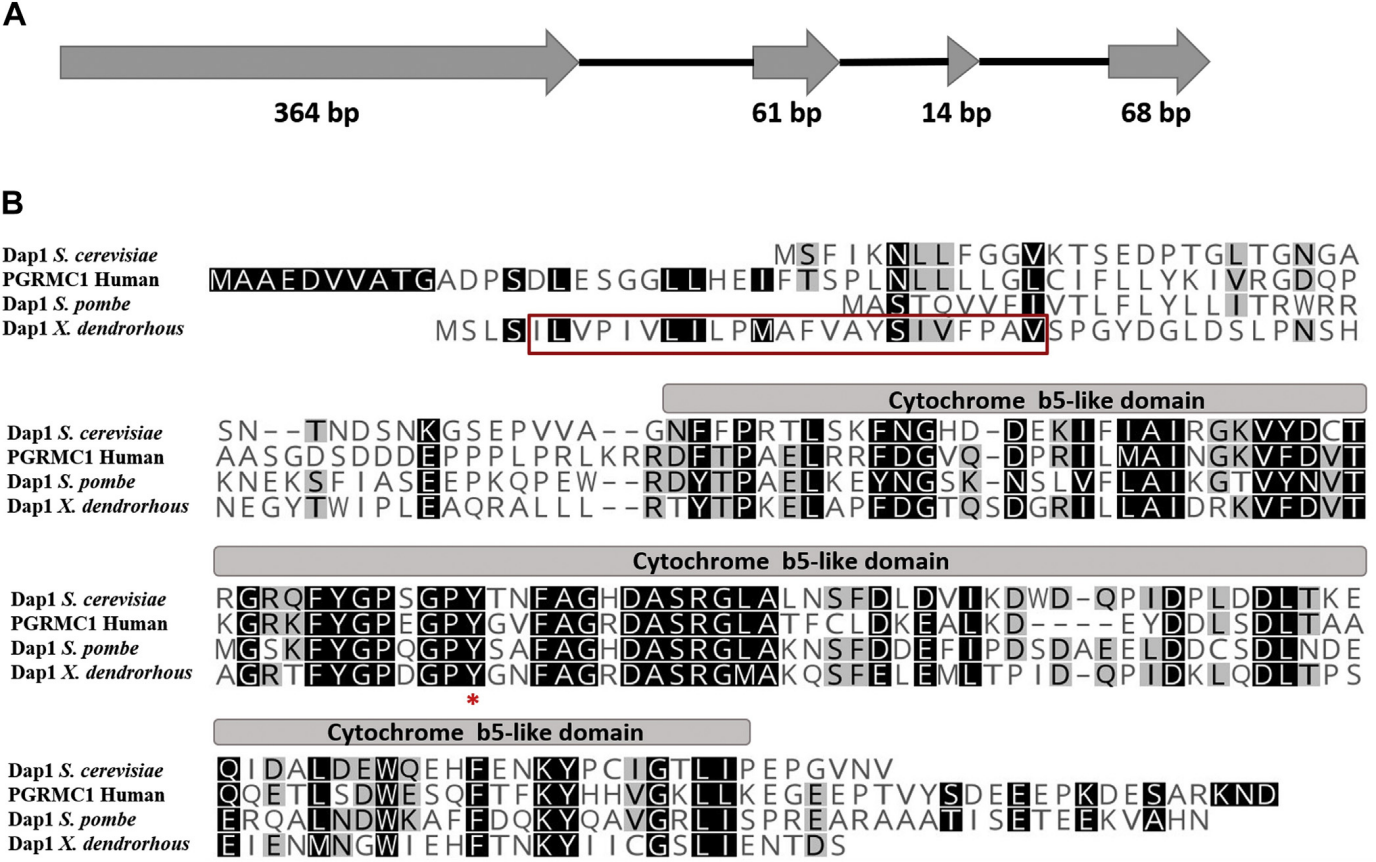
*Xanthophyllomyces dendrorhous DAP1* gene structure and sequence alignment. A: *DAP1* gene structure, with exons represented by gray arrows; the size is indicated under each exon. B: Protein sequence alignment of PGRMC1 from *H. sapiens* (NP_006658.1) and Dap1 from the yeasts *Saccharomyces cerevisiae* (NP_015155.1) and *Schizosaccharomyces pombe* (NP_594461.1). The red box encloses the predicted transmembrane segment, and the conserved tyrosine that coordinates heme is indicated with a red asterisk.

Using bioinformatic analyses of genomic and transcriptomic data from *X. dendrorhous* (35), we identified the putative *X. dendrorhous DAP1* gene (GenBank: MN956832). From analyses of the genomic DNA and complementary DNA sequences, the gene structure of *DAP1* was determined. The *DAP1* gene from *X. dendrorhous* comprises four exons (exon 1, 364 bp; exon 2, 61 bp; exon 3, 14 bp; and exon 4, 68 bp) (Fig. 2A) and three introns (123, 77, and 101 bp in size), accounting for 808 bp from the translation initiation site to the translation stop codon and a coding region of 507 bp. This gene encodes a predicted Dap1 protein of 168 amino acids with a molecular weight of 18.62 kDa (pI [isoelectric point] = 4.30). Bioinformatic analysis of the Dap1 protein identified a transmembrane segment in its N-terminus followed by a CYB5-like domain, with a conserved tyrosine at position 96 (Fig. 2B). According to previous observations, this tyrosine is essential for coordination of the heme group in this type of protein (36, 37). According to Kabe *et al.* (37), in contrast to the CYB5 protein, the heme iron is six-coordinated by two axial histidine residues, and in PGRMC1, the heme iron is five-coordinated by tyrosine, as determined by X-ray crystallography.

### *DAP1* gene mutation in *X. dendrorhous*

**Fig. 3:**
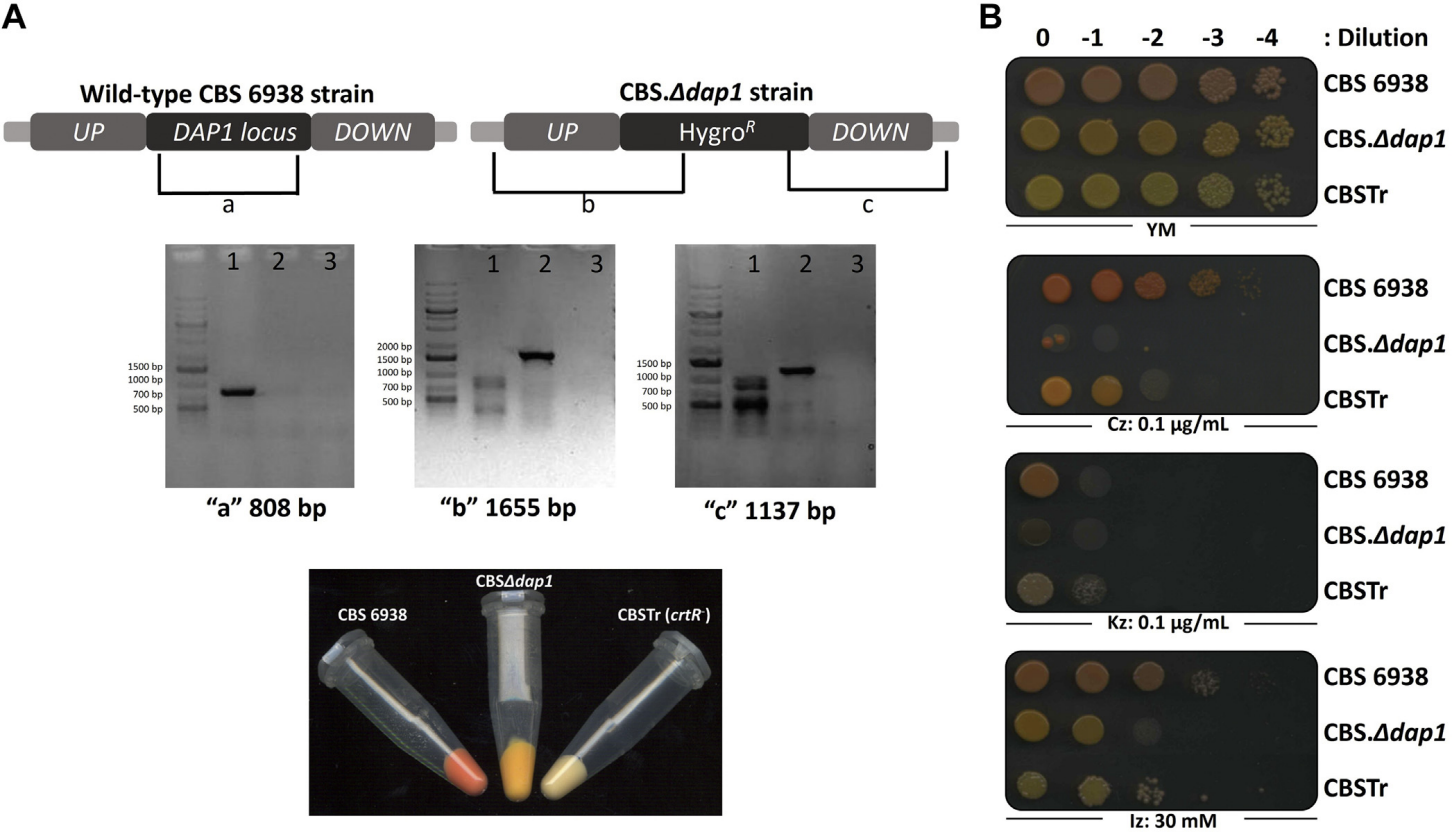
Generation, analysis, and phenotype of strain CBS.*Δdap1*. A, Top: Scheme of the *DAP1* locus in strains CBS 6939 (wild type) and CBS.*Δdap1*. Fragments “a,” “b,” and “c” represent the amplified fragments used in PCR analyses to confirm *DAP1* gene replacement with a hygromycin B resistance cassette (Hygro^*R*^) through a double homologous recombination event. The amplification of “a” was expected in only the wild-type strain and that of “b” and “c” was expected in strain CBS.*Δdap1*. Middle: Amplification of fragments “a,” “b,” and “c” in strains CBS 6938 (lane 1) and CBS.*Δdap1* (lane 2) and a negative control (lane 3). The expected sizes of fragments “a,” “b,” and “c” are indicated under each gel. The GeneRuler 1 kb Plus DNA Ladder was used as a molecular weight marker. Bottom: Cell pellets of strains CBS 6939, CBS.*Δdap1*, and CBSTr. B: Five microliter microdrops of cultures of *Xanthophyllomyces dendrorhous* strains CBS 6939, CBS.*Δdap1*, and CBSTr serially diluted by 0 to −4 (dilutions 0–10^−4^) were seeded on YM agar plates (1.5%) with different supplements and incubated for 5 days at 22°C. From top to bottom: control YM medium and YM medium supplemented with clotrimazole (Cz, 0.1 μg/ml), ketoconazole (Kz, 0.1 μg/ml), or itraconazole (Iz, 30 mM).

To examine whether the *DAP1* gene is required for the synthesis of ergosterol and astaxanthin in *X. dendrorhous*, a deletion mutant was generated by replacing the *DAP1* gene with a module that confers resistance to hygromycin B to *X. dendrorhous* through homologous recombination in the parental strain CBS 6938. Generation of the designed mutant, CBS.*Δdap1*, was confirmed by PCR analysis using a specific set of primers (Fig. 3A). Visually, CBS.*Δdap1* and the wild-type strain were found to exhibit different color phenotypes, as the first was more orange than the second when cultured in YM medium. Strain CBSTr served as control as its *crtR* gene, which encodes a CPR that interacts with P450s by donating electrons necessary for the activity of the P450s, is disrupted. CBSTr is yellow as it is unable to produce astaxanthin and accumulates β-carotene (17). In addition, the ergosterol fraction is reduced in strain CBSTr compared with the wild-type strain, and strain CBSTr accumulates other unidentified sterols (18). Considering that the *DAP1* gene may be required for the synthesis of ergosterol and that the growth of *dap1*^*−*^ mutants from *S. pombe* (8), *S. cerevisiae* (38), and *Aspergillus fumigatus* (39) was affected by supplementation of the medium with azoles as these drugs inhibit sterol biosynthesis, this parameter was also evaluated. The growth of both analyzed *X. dendrorhous* mutants, CBS.*Δdap1* and CBSTr, was weaker than that of the wild-type strain when cultured in the presence of the azoles clotrimazole, ketoconazole, or itraconazole; interestingly, CBS.*Δdap1* was more sensitive than CBSTr to these drugs, suggesting that ergosterol biosynthesis in the CBS.Δ*dap1* mutant was also affected (Fig. 3B).

Next, the effect of the *DAP1* deletion on carotenoid and sterol production was evaluated. For comparative purposes, the three strains, CBS 6938, CBS.*Δdap1*, and CBSTr, were cultured in triplicate in YM medium with constant agitation at 22°C until reaching the stationary phase of growth. In general, the *Δdap1* mutation did not affect growth under the studied conditions, as the three strains showed similar growth curves (supplemental Fig. S2). After 120 h of culture, samples were taken to extract carotenoids and sterols, which were quantified spectrophotometrically, and carotenoid and sterol composition was analyzed by RP-HPLC. The total carotenoid content from strain CBS.*Δdap1* was similar to that from the wild-type strain, but the composition of the carotenoids differed (Table 2). The wild-type CBS 6938 strain produces mainly astaxanthin (approximately 82%), unlike the CBSTr strain, which does not produce astaxanthin as it does not have a functional CPR to donate electrons to the P450 CrtS (17) and thus mainly accumulates β-carotene (approximately 88%). Interestingly, the CBS.*Δdap1* strain showed an intermediate phenotype as it still produced astaxanthin, but the astaxanthin proportion was drastically reduced to approximately 3%, and this strain accumulated mainly the substrate of the P450 CrtS, β-carotene (approximately 58%). A similar pattern was observed when sterols were analyzed: no significant differences in the total sterol content were observed among strains, but the strains showed different sterol compositions (Table 2). The wild-type strain produced ergosterol as the main sterol. In contrast, the ergosterol fraction decreased to approximately 80% in strain CBSTr, which accumulated two other unidentified metabolites with a sterol spectrum, hereinafter referred to as peak 1_(12min)_ and peak 2_(17.2min)_ according to their approximate retention time. As in CBSTr, the ergosterol fraction in strain CBS.*Δdap1* was decreased but to a greater extent (less than 10%), and this strain also accumulated two other unidentified metabolites with a sterol spectrum with the same retention times as strain CBSTr. Sterol samples from strains CBS.*Δdap1* and CBSTr were mixed and coinjected in the HPLC apparatus; three peaks were observed, indicating that both strains probably produce the same three metabolites but in different proportions.

**Table 2:** Production and composition of carotenoids and sterols in strains CBS 6938, CBS.*Δdap1*, and CBSTr

Metabolites	Strains		
	CBS 6938	CBS.*Δdap1*	CBSTr
Sterols (mg/g)			
Ergosterol	2.5 ± 0.01^a^	0.1 ± 0.03^b^	2.1 ± 0.05^c^
Peak 1_(12min)_	ND	0.5 ± 0.02^a^	0.3 ± 0.02^b^
Peak 2_(17.2min)_	ND	1.4 ± 0.01^a^	0.2 ± 0.03^b^
Total sterols	2.5 ± 0.3^a^	2.1 ± 0.3^a^	2.6 ± 0.2^a^
Carotenoids (μg/g)			
Astaxanthin	574.0 ± 15.9^a^	16.8 ± 2.9^b^	ND
Astaxanthin intermediates	61.8 ± 6.4^a^	124.8 ± 3.9^b^	51.6 ± 2.1^a^
β-carotene	19.2 ± 4.6^a^	354.2 ± 4.8^b^	675.5 ± 2.9^c^
Other carotenoids	46.0 ± 14.2^a^	115.1 ± 10.5^b^	41.5 ± 6.6^a^
Total carotenoids	701.1 ± 47.2^a^	610.9 ± 59.4^a^	768.5 ± 47.4^a^

ND, not detected.

Total sterols and carotenoids were extracted after 120 h of culture and normalized to the yeast dry weight in grams. The table shows the mean ± standard deviation of three independent cultures of each strain. Peaks 1 and 2 correspond to metabolites with a sterol spectrum that were observed in chromatograms after approximately 12.0 and 17.2 min of retention time, respectively. Intermediary carotenoids from β-carotene to astaxanthin include phoenicoxanthin, hydroxyechinenone, and echinenone; other carotenoids include torulene, hydroxyketotorulene, and other unidentified carotenoids. Data were evaluated with one-way ANOVA and the Tukey post hoc test to compare metabolite production between strains. Superscripted letters indicate statistical comparisons: the use of the same letter denotes no statistically significant differences, and the use of different letters indicates a significant difference between strains with *P* < 0.01.

In summary, the *Δdap1* mutation affected the synthesis of sterols and carotenoids in *X. dendrorhous* by affecting their composition and had a greater impact than the *crtR*^*−*^ mutation, since its ergosterol fraction was reduced by a much greater extent than that of the *crtR*^*−*^ mutant.

### Hemin suppresses a growth defect in clotrimazole in CBS.*Δdap1*

**Fig. 4:**
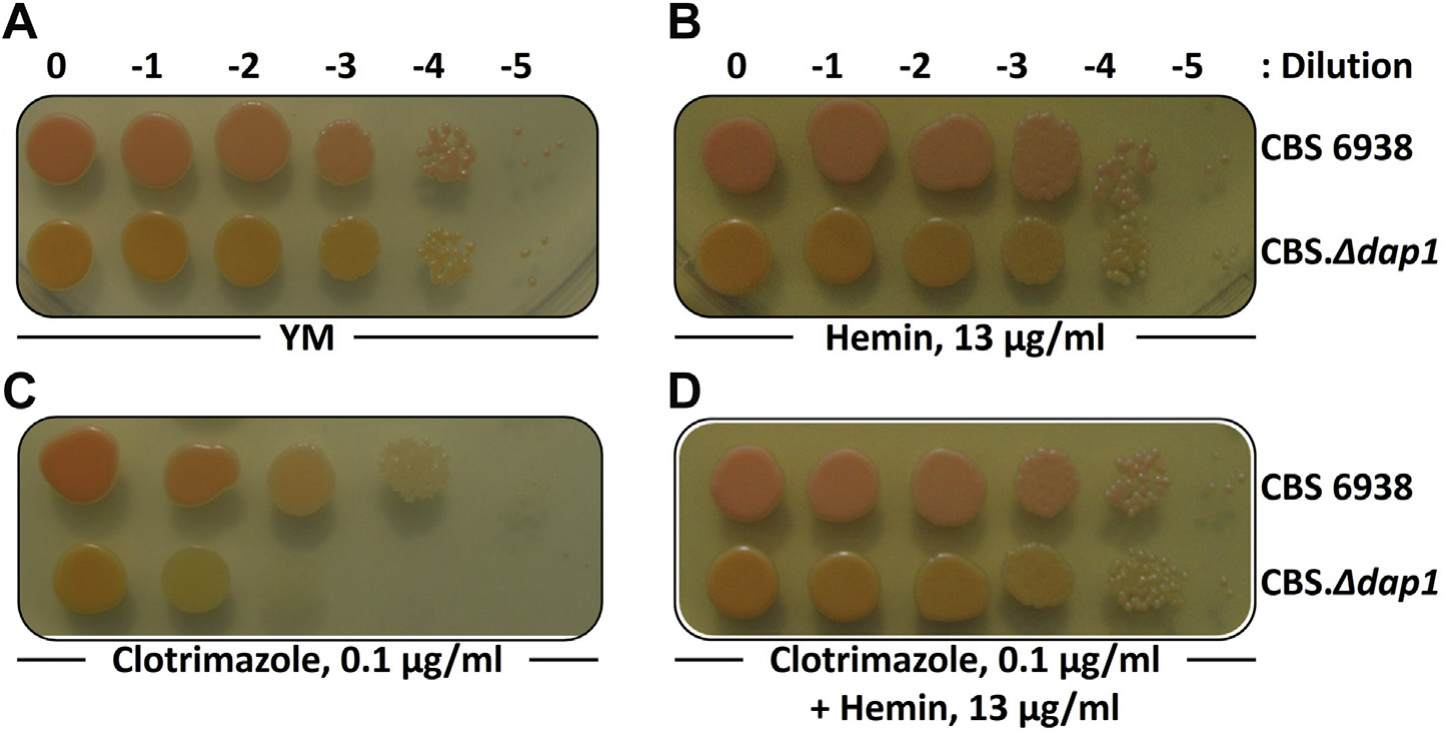
The addition of hemin reverts the clotrimazole sensitivity phenotype of strain CBS.*Δdap1*. Five microliter microdrops of cultures of *Xanthophyllomyces dendrorhous* strains CBS 6939 (wild-type) and CBS.*Δdap1* serially diluted 0 to −5 (dilutions 0–10^−5^) were seeded on YM agar plates (1.5%) with different supplements and incubated for 5 days at 22°C. A: Control (YM medium) and YM medium supplemented with (B) hemin (13 μg/ml), (C) clotrimazole (0.1 μg/ml), and (D) clotrimazole (0.1 μg/ml) and hemin (13 μg/ml).

P450 enzymes are hemoproteins, so they require the heme prosthetic group for their activity. In *S. cerevisiae*, the exogenous addition of hemin to *Δdap1* mutants partially restored the attenuated Cyp51 function observed in these mutants (11). Therefore, we evaluated whether the exogenous addition of hemin was sufficient to reverse the color and clotrimazole-sensitivity phenotypes observed in CBS.*Δdap1*. For this, microdrops of serially diluted cultures of the wild-type and CBS.*Δdap1* strains were seeded on YM medium plates supplemented with 13 μg/ml hemin, 0.1 μg/ml clotrimazole, or both (Fig. 4). As indicated before, the growth of CBS.*Δdap1* in YM agar medium was not affected, and the colonies were more orange than those of the wild-type strain (Fig. 4A). Supplementation with hemin did not affect the growth or color phenotype (Fig. 4B), and CBS.*Δdap1* was shown to be more sensitive to clotrimazole than the wild-type strain (Fig. 4C). Interestingly, simultaneous supplementation with hemin reversed the clotrimazole sensitivity phenotype observed in CBS.*Δdap1* but did not affect its color phenotype (Fig. 4D). Even though hemin was supplemented at up to 90 μg/ml, no visual change in color phenotype in CBS.*Δdap1* was observed (data not shown). Thus, the addition of hemin to CBS.*Δdap1* cultures reversed the clotrimazole sensitivity phenotype but had no apparent effect on the color phenotype.

Considering that growth on azoles may be a more sensitive test than a qualitative evaluation of color phenotype, the effect of hemin supplementation on carotenoid and sterol production was quantified. The wild-type and CBS.*Δdap1* strains were cultured in YM medium with or without 13 μg/ml hemin supplementation. As a point of consensus, carotenoids and sterols were extracted at the exponential phase of growth, as major differences in sterol production were observed at the early phases of growth in *X. dendrorhous*, but major differences in carotenoid production were observed at the late phases of growth in *X. dendrorhous* (Table 3). No significant differences were observed in the total content of either type of metabolite among strains or among conditions (with or without hemin supplementation). Even though there was no statistically significant difference when the carotenoid composition was evaluated, a trend in which the β-carotene fraction was reduced while the sum of astaxanthin and intermediary carotenoids from β-carotene to astaxanthin (all CrtS products) was increased was observed in CBS.*Δdap1* cultures supplemented with hemin. The absence of significant differences in the composition of carotenoids when hemin was added could be associated with the measurement of these metabolites in the exponential phase and not in the stationary phase of growth, when mainly carotenoids have accumulated (40). Different sterol compositions were observed in CBS.*Δdap1* cultures supplemented with hemin compared with cultures without supplementation with this compound. Hemin supplementation decreased the peak 2_(17.2min)_ fraction, and three additional unidentified metabolites with a sterol spectrum (peak 3_(12.6min)_, peak 4_(14.5min)_, and peak 5_(18.2min)_) were detected. These unidentified metabolites detected with hemin supplementation could mediate the clotrimazole resistance phenotype, as shown in Fig. 4.

**Table 3:** Composition and production of sterols and carotenoids in *X. dendrorhous* strains in cultures with (+H) or without hemin supplementation

Metabolites	Strains			
	CBS 6938	CBS 6938 (+H)	CBS.*Δdap1*	CBS.*Δdap1* (+H)
Sterols (mg/g)				
Ergosterol	2.9 ± 0.01^a^	2.7 ± 0.01^a^	0.2 ± 0.02^b^	0.3 ± 0.03^b^
Peak 1_(12min)_	ND	ND	1.1 ± 0.11^a^	1.1 ± 0.09^a^
Peak 2_(17.2min)_	ND	ND	2.4 ± 0.09^a^	1.8 ± 0.14^b^
Peak 3_(12.6min)_	ND	ND	ND	0.2 ± 0.04
Peak 4_(14.5min)_	ND	ND	ND	0.06 ± 0.01
Peak 5_(18.2min)_				0.4 ± 0.12
Total sterols	3.0 ± 0.7^a^	2.7 ± 0.04^a^	3.8 ± 0.6^a^	3.8 ± 0.3^a^
Carotenoids (μg/g)				
Astaxanthin	510.7 ± 39.4^a^	452.1 ± 21.1^a^	15.3 ± 4.5^b^	14.4 ± 10.8^b^
Astaxanthin intermediates	325.1 ± 60.1^a^	315.7 ± 51.5^a^	418.5 ± 38.6^a^	514.1 ± 78.3^a^
β-carotene	122.8 ± 27.0^a^	172.3 ± 21.4^a^	656.9 ± 32.8^b^	712.0 ± 83.8^b^
Other carotenoids	36.6 ± 15.4^a^	32.6 ± 15.5^a^	23.6 ± 12.6^a^	32.0 ± 19.0^a^
Total carotenoids	995.0 ± 320.6^a^	972.7 ± 109.4^a^	1118.4 ± 407.3^a^	1272.6 ± 183.6^a^

ND, not detected.

Total sterols and carotenoids were extracted after 120 h of culture and normalized to the yeast dry weight in grams. The table shows the mean ± standard deviation of three independent cultures of each strain. Peaks 1, 2, 3, 4, and 5 correspond to metabolites with a sterol spectrum that were observed in chromatograms after approximately 12.0, 17.2, 12.6, 14.5, and 18.2 min of retention time, respectively. Intermediary carotenoids from β-carotene to astaxanthin include phoenicoxanthin, hydroxyechinenone, and echinenone; other carotenoids include torulene, hydroxyketotorulene, and other unidentified carotenoids. Data were evaluated with one-way ANOVA and the Tukey post hoc test to compare metabolite production between strains. Superscripted letters indicate statistical comparisons: the use of the same letter denotes no statistically significant differences, and the use of different letters indicates a significant difference between strains with *P* < 0.01.

### Expression levels of genes encoding P450 systems in CBS.*Δdap1*

**Fig. 5:**
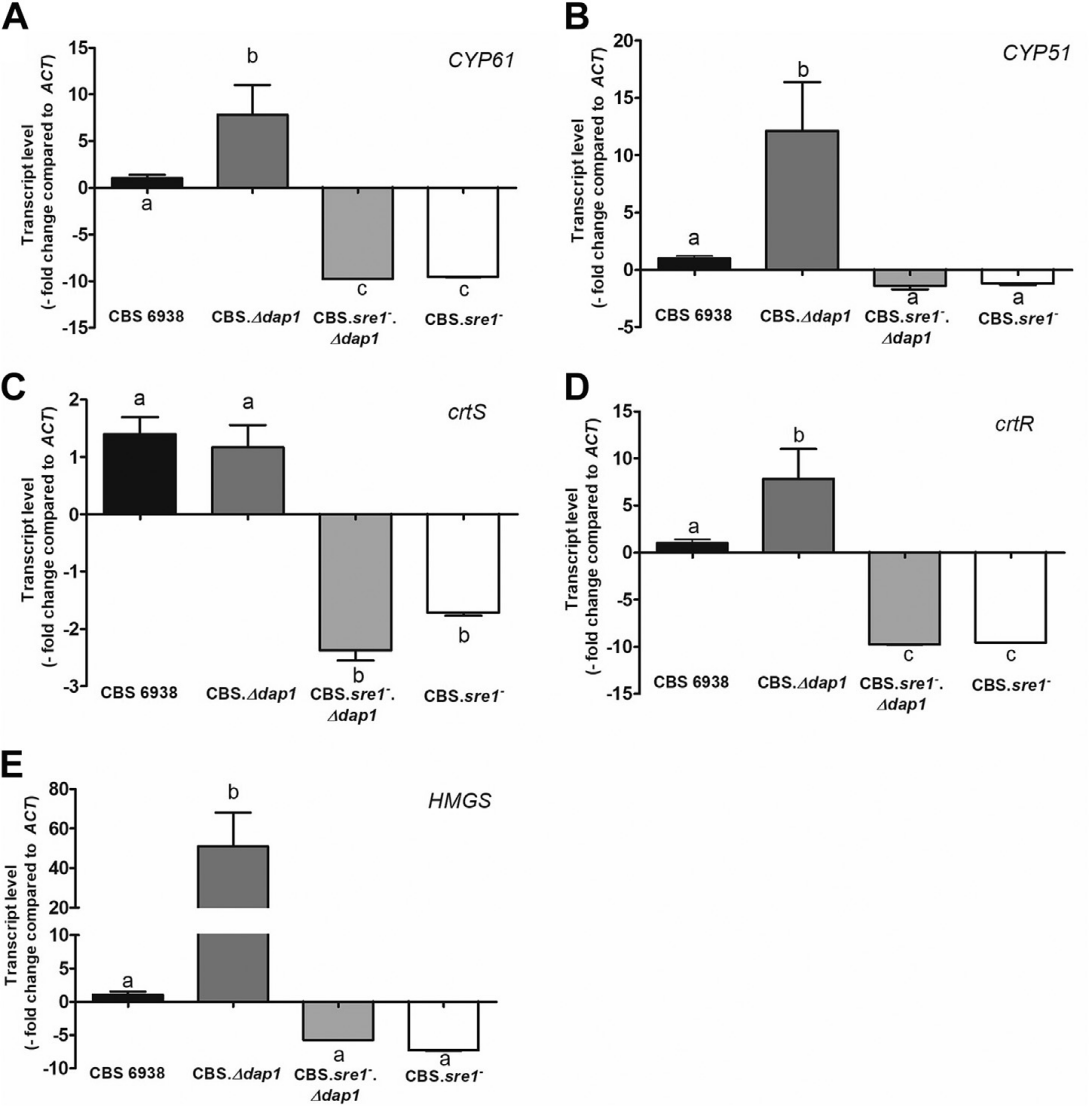
Relative transcript levels of genes of the P450 systems in strains CBS 6938, CBS.*Δdap1*, CBS.*sre1*^*−*^*.Δdap1*, and CBS.*sre1*^*−*^*.* The relative transcript levels of the genes (A) *CYP61* (GenBank: JX183236), (B) *CYP51* (GenBank: KP317478), (C) *crtS* (GenBank: DQ202402.1), (D) *crtR* (GenBank: EU884133), and (E) *HMGS* (GenBank: MK368600) after 120 h of culture were evaluated by RT-qPCR and normalized to the housekeeping gene β-actin (GenBank: X89898.1). Values are the mean ± standard deviation of three independent cultures (one-way ANOVA and Tukey post hoc test; different letters indicate significant differences with *P* < 0.01).

The results of carotenoid and sterol analysis in the CBS.*Δdap1* strain strongly suggest that steps catalyzed by P450 systems in both metabolic pathways are affected. To evaluate whether this effect occurs at the transcriptional level, transcript levels of the P450 genes *CYP51* and *CYP61* in ergosterol biosynthesis and *crtS* in astaxanthin biosynthesis were analyzed by RT-qPCR (Fig. 5). In addition, transcript levels of the *crtR* gene were evaluated, as this gene encodes a P450 reductase that is involved in both biosynthetic pathways (18). Interestingly, even though the production of both end products, ergosterol and astaxanthin, was reduced in the CBS.*Δdap1* strain, the transcript levels of most of the P450 system genes involved in their synthesis were higher compared with those in the wild-type strain (the *CYP61*, *CYP51*, and *crtR* genes). These results support the hypothesis that Dap1 does not act at the transcriptional level.

**Fig. 6:**
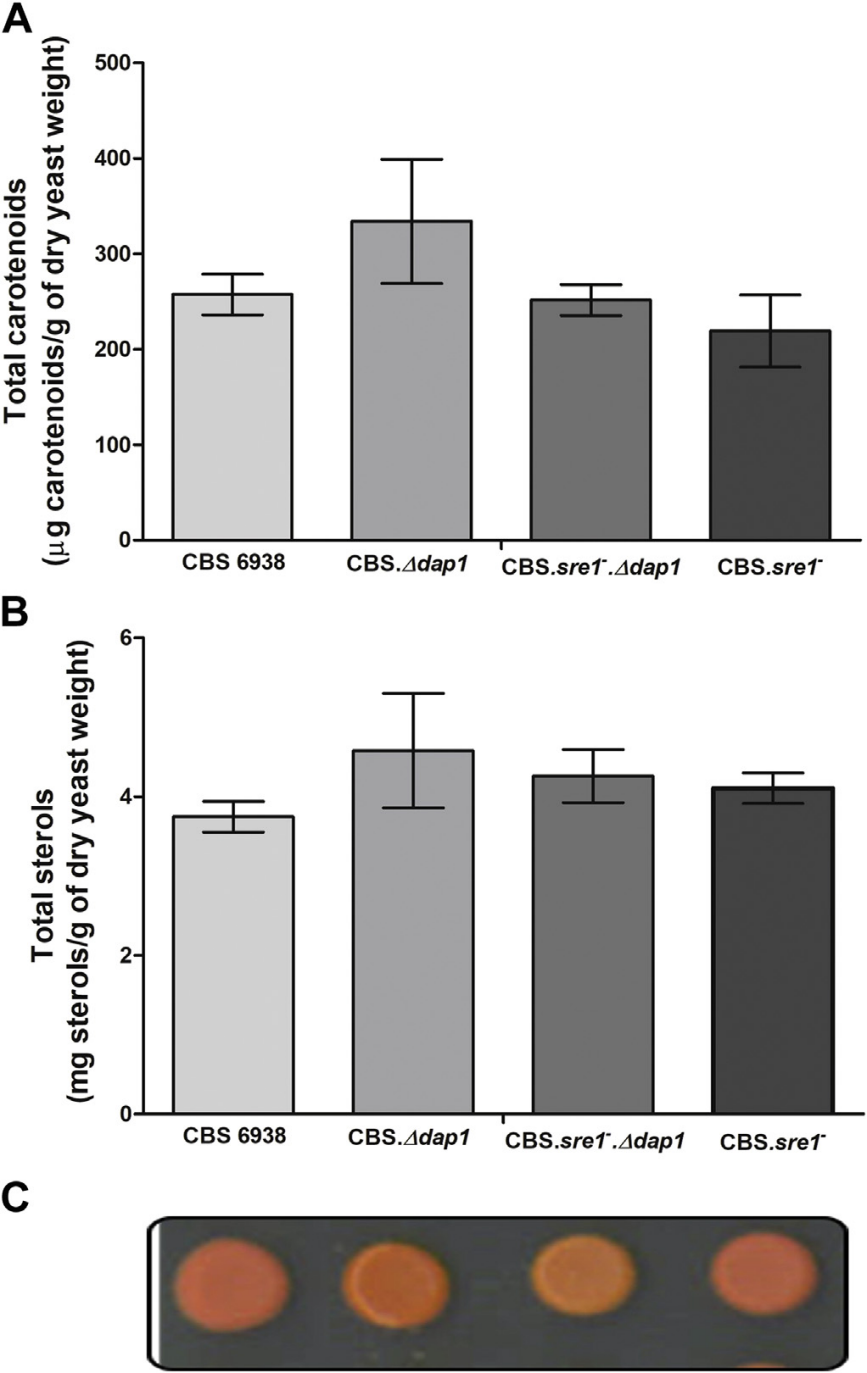
Production of carotenoids and sterols in strains CBS 6938, CBS.*Δdap1*, CBS.*sre1*^*−*^*.Δdap1*, and CBS.*sre1*^*−*^*.* A: Carotenoid production is expressed as microgram of carotenoids/g of dry yeast weight. B: Sterol production is expressed as milligram of sterols/g of dry yeast weight. The mean ± standard deviation of three independent cultures of each strain is shown. One-way ANOVA followed by the Tukey post-test was used as a statistical test. No significant differences were observed among strains. C: Color phenotype of each strain.

In *S. pombe*, expression of the *DAP1* gene is regulated by the transcription factor Sre1 (8), which regulates sterol homeostasis in cells. In general, Sre1 is activated when sterol levels decrease, inducing the expression of genes involved in the synthesis of sterols. Potential Sre1-binding sites have been identified in the promoter regions of the *X. dendrorhous* genes *CYP51*, *CYP61* (13), and *crtR*, and the Sre1-encoding gene of *X. dendrorhous* was recently characterized (20, 41), and genes *CYP61* and *crtR* were confirmed as direct Sre1 targets by chromatin immunoprecipitation-exo analysis (35). Considering that some genes encoding P450 systems in *X. dendrorhous* are under the regulation of Sre1 and that sterol composition was dramatically changed in strain CBS.*Δdap1* (a condition that might activate Sre1, which could be responsible for the higher *CYP61*, *CYP51*, and *crtR* transcript levels observed in strain CBS.*Δdap1*), strain CBS.*sre1*^*−*^*.Δdap1* was constructed in the same way that strain CBS.*Δdap1* was obtained. Strain CBS.*sre1*^*−*^*.Δdap1* was constructed from strain CBS.*sre1*^*−*^ (20), which in turn was derived from CBS 6938. Then, gene transcript levels were evaluated. As a control, the *HMGS* gene of the mevalonate pathway, which generates the precursors for the biosynthesis of sterols and carotenoids, was also included, as it was shown that Sre1 could directly bind the promoter region of this gene (20, 35). In strain CBS.*sre1*^*−*^, relative transcript levels of the genes *CYP61*, *crtS*, *crtR*, and *HMGS* were lower than those in the wild-type strain (Fig. 5). In strain CBS.*sre1*^*−*^*.Δdap1*, the transcript levels of all the evaluated genes were similar to those in strain CBS.*sre1*^*−*^. Strain CBS.*sre1*^*−*^ exhibited the color phenotype of the wild-type strain, but double-mutant CBS.*sre1*^*−*^*.Δdap1* colonies were more orange than wild-type colonies and even lighter than in CBS.*Δdap1* colonies, suggesting a decrease in the astaxanthin fraction and an increase in the β-carotene fraction in this strain (Fig. 6). To evaluate this possibility, total carotenoids and sterols were extracted and quantified, and no significant differences in their abundance were detected among strains (Fig. 6). Even though no statistically significant differences in the proportion of ergosterol and astaxanthin were found between strains CBS.*Δdap1* and CBS.*sre1*^*−*^*.Δdap1* when sterol and carotenoid composition was analyzed (Table 4), a tendency was observed: the fraction of both end products was reduced in strain CBS.*sre1*^*−*^*.Δdap1* (ergosterol: 10.4%–5.7%, astaxanthin: 3.9%–2.3%, approximately). The same tendency was observed in several experimental replicates. Moreover, the proportion of the unidentified metabolites with a sterol spectrum, peak 1_(12min)_ and peak 2_(17.2min)_, was significantly different in strains CBS.*sre1*^*−*^*.Δdap1* and CBS.*Δdap1*, and the fraction of the sum of oxygenated intermediary carotenoids in the synthesis of astaxanthin from β-carotene by CrtS, such as echinenone, hydroxyechinenone, and phoenicoxanthin, was significantly reduced in strain CBS.*sre1*^*−*^*.Δdap1* compared with strain CBS.*Δdap1*. These results suggest that the SREBP pathway is activated in strain CBS.*Δdap1*, probably because of changes in sterol composition, but the activation of this pathway is not sufficient to maintain wild-type sterol and carotenoid levels in strain CBS.*Δdap1*.

**Table 4:** Production of sterols and carotenoids in strains CBS 6938, CBS.*Δdap1*, CBS.*sre1*, and CBS.sre1^*−*^*Δdap1*

Metabolites (%)	Strains			
	CBS 6938	CBS.*Δdap1*	CBS.*sre1*^*−*^	CBS.*sre1*^*−*^.*Δdap1*
Sterols				
Ergosterol	97.1 ± 1.8^a^	10.4 ± 2.1^b^	99.2 ± 0.3^a^	5.7 ± 2.2^b^
Peak 1_(12min)_	ND	27.2 ± 8.0^a^	ND	8.3 ± 3.8^b^
Peak 2_(17.2min)_	ND	62.5 ± 6.0^a^	ND	86.1 ± 1.6^b^
Carotenoids				
Astaxanthin	63.9 ± 3.7^a^	3.9 ± 2.1^b^	50.3 ± 12.8^a^	2.3 ± 0.3^b^
Intermediary carotenoids from β-carotene to astaxanthin	23.9 ± 2.7^a^	41.3 ± 3.4^b^	34.4 ± 5.9^a^	35.5 ± 0.6^a^
β-carotene	5.4 ± 0.9^a^	50.0 ± 6.8^b^	8.4 ± 4.2^a^	58.6 ± 1.7^b^
Other carotenoids	6.8 ± 0.8^a^	4.9 ± 0.7^a,b^	6.8 ± 0.9^a^	3.7 ± 0.3^b^

ND, not detected.

Total sterols and carotenoids were extracted after 120 h of culture and normalized to the yeast dry weight in grams. The table shows the mean ± standard deviation of three independent cultures of each strain. Peaks 1 and 2 correspond to metabolites with a sterol spectrum that were observed in chromatograms after approximately 12.0 and 17.2 min of retention time, respectively. Intermediary carotenoids from β-carotene to astaxanthin include phoenicoxanthin, hydroxyechinenone, and echinenone; other carotenoids include torulene, hydroxyketotorulene, and other unidentified carotenoids. Data were evaluated with one-way ANOVA and the Tukey post hoc test to compare metabolite production between strains. Superscripted letters indicate statistical comparisons: the use of the same letter denotes no statistically significant differences, and the use of different letters indicates a significant difference between strains with *P* < 0.01.

### Interaction of Dap1 with P450s from *X. dendrorhous*

Our data support that Dap1 works with each of the three characterized P450s in *X. dendrorhous*. To evaluate the potential interaction of Dap1 with P450s from *X. dendrorhous*, co-IP experiments were designed. First, strains synthesizing proteins fused to standard epitopes were constructed (supplemental Fig. S1). The Dap1 protein was fused to the FLAG epitope at its C-terminal end (Dap1-3xFLAG), and the P450s CrtS, Cyp61, and Cyp51 were fused to the HA epitope at their C-terminal end (CrtS-3xHA, Cyp61-3xHA, and Cyp51-3xHA, respectively). Several vectors were constructed by DNA assembler in *S. cerevisiae* (23, 24) to then replace the native *X. dendrorhous* genes through homologous recombination with the version of the gene that encodes the corresponding fusion protein. In this way, four strains were generated: *i*) CBS.*DAP1.FLAG*, *ii*) CBS.*DAP1.FLAG-crtS.HA*, *iii*) CBS.*DAP1.FLAG-CYP61.HA*, and *iv*) CBS.*DAP1.FLAG-CYP51.HA*, which were all confirmed by PCR analyses using specific sets of primers. Visually, all strains had the same phenotype as the wild-type strain CBS 6938, and no differences in the amounts of total carotenoids and sterols were detected (supplemental Fig. S3), indicating that the introduced modifications did not affect the corresponding protein function.

**Fig. 7:**
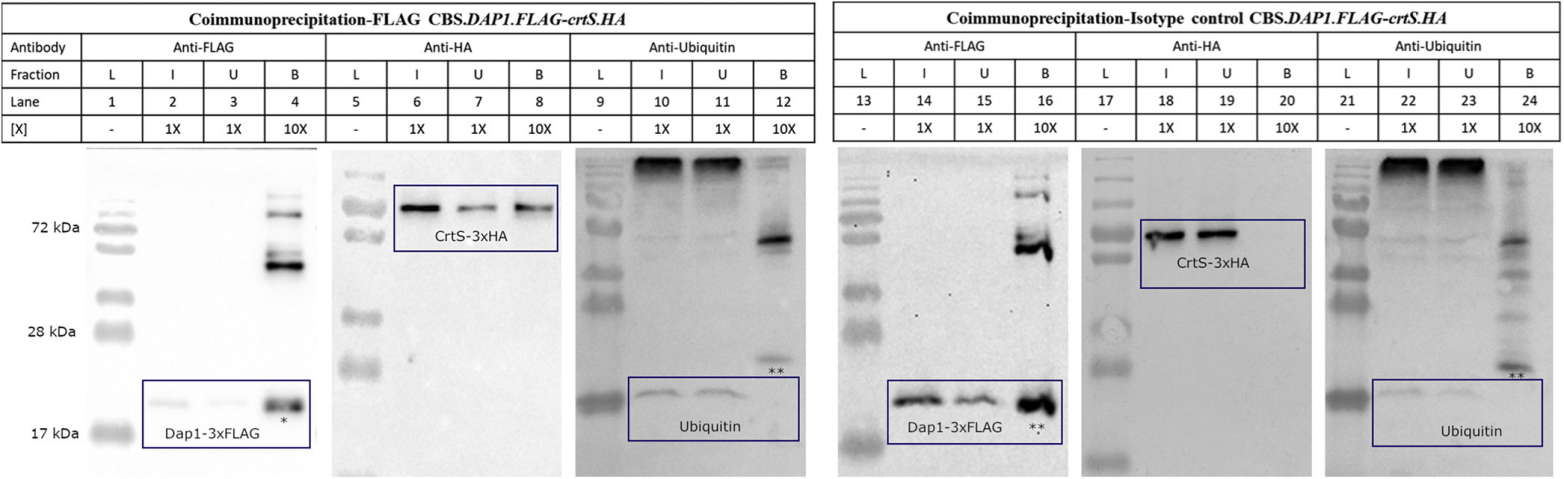
Coimmunoprecipitation of Dap1 with CrtS. Protein extracts from CBS.*DAP1.FLAG-crtS.HA* strain were subjected to immunoprecipitation with anti-FLAG antibody or mouse IgG1 kappa monoclonal as an isotype control. The input (I), unbound (U), and bound (B) fractions were analyzed by Western blotting with anti-FLAG (Dap1-3xFLAG immunoprecipitation control), anti-HA (to evaluate the coimmunoprecipitation of CrtS-3xHA with Dap1-3xFLAG), or antiubiquitin (as a coimmunoprecipitation specificity control) antibodies. In Western blotting with anti-FLAG and antiubiquitin, anti-mouse IgG H&L-Peroxidase was used as the secondary antibody (anti-HA fused to peroxidase). The expected size of the proteins was 21.3, 66.1, and 16.8 kDa for Dap1-3xFLAG, CrtS-3xHA, and ubiquitin, respectively: the blue boxes frame the position of the analyzed bands in each gel. ∗ Indicates a double band (one corresponding to the Dap1-3xFLAG protein [higher band] and the other to the light chain of the antibody used for immunoprecipitation[(lower band]), and ∗∗ indicates a single band corresponding to the light chain of the antibody used for immunoprecipitation. The bound fraction is 10X more concentrated than the input and unbound fractions. PageRuler Plus 10–250 kDa was used as a molecular weight standard (L).

Co-IP experiments were performed with an anti-FLAG antibody to immunoprecipitate Dap1-3xFLAG or mouse IgG1 kappa monoclonal as an isotype control using protein extracts from the wild-type and constructed strains (Fig. 7). After immunoprecipitation, the input, unbound, and bound fractions were resolved by SDS-PAGE, and the presence of specific proteins in each fraction was detected by Western blotting using three antibodies: *i*) anti-FLAG to detect Dap1-3xFLAG; *ii*) anti-HA to detect the P450 enzyme Cyp61/Cyp61/CrtS-3xHA; and *iii*) antiubiquitin to detect ubiquitin as a Dap1 co-IP negative control (the bound fraction contains the light and heavy chains of the antibody used for immunoprecipitation, which were recognized by the secondary antibody: anti-mouse IgG-HRP, used in Western blotting with anti-FLAG and antiubiquitin). As expected, FLAG-fusion protein or HA-fusion protein was not detected in any fraction using the wild-type CBS 6938 protein extract in the anti-FLAG immunoprecipitation assay (supplemental Fig. S4A); ubiquitin was detected in the input and unbound fractions but not in the bound fraction. In this strain, only the light and heavy chains of the antibody used for immunoprecipitation (anti-FLAG and antiubiquitin) are observed in the bound fractions (supplemental Fig. S4A). When the assay was performed using protein extracts from strain CBS.*DAP1.FLAG*, only Dap1-3xFLAG, but not the HA-fusion proteins, was detected (supplemental Fig. S4B). When the same protein extract was used, Dap1-3xFLAG was not detected in the bound fraction of the co-IP isotype control assay, confirming that anti-FLAG antibody used for immunoprecipitation was specific to the Dap1-3xFLAG target (supplemental Fig. S4B). As a result, CrtS-3xHA was detected in the bound fraction (Fig. 7, lane 8) when the assay was performed using protein extract from CBS.*DAP1.FLAG-crtS.HA* strain, and ubiquitin was not detected in the bound fraction (Fig. 7, lane 12). The HA-labeled CrtS was not detected at the bound fraction in the isotype co-IP control assay (Fig. 7, lane 20). These results indicate that CrtS coimmunoprecipitates with Dap1. Under the same experimental conditions, co-IP assays were performed using the strains CBS.*DAP1.FLAG-CYP61.HA* and CBS.*DAP1.FLAG-CYP51.HA*, obtaining the same results as for CrtS-3xHA: Cyp61-3xHA and Cyp51-3xHA coimmunoprecipitates with Dap1-3XFLAG (supplemental Fig. S5A, B). In summary, the three P450s under study coimmunoprecipitated with Dap1-3xFLAG, Dap1 specifically bound P450s, but not ubiquitin.

## Discussion

A new role for the Dap1 protein was identified in this work. In the carotenogenic yeast *X. dendrorhous*, the specificity of CrtS by the electron donor CrtR was previously reported, as astaxanthin was produced in *S. cerevisiae* only when the *X. dendrorhous crtS* gene was coexpressed with *crtR* (42). Our current work demonstrates the ability of CrtS to interact with another protein: Dap1. According to bioinformatic analyses, the identified *DAP1* gene from *X. dendrorhous* encodes a protein with a transmembrane segment in its N-terminal portion and a CYB5-like domain in its C-terminal portion, which would allow it to bind heme, as has been described in other organisms (36). The CYB5-like domain has a conserved tyrosine that mediates this interaction. According to our results, the deletion of *DAP1* (strain CBS.*Δdap1*) changed yeast pigmentation, indicating that carotenogenesis was altered in this mutant. The altered step was that catalyzed by CrtS, as the substrate of this enzyme, β-carotene, accumulated in the *Δdap1* strain. In contrast to the phenotype of strain CBSTr (a *crtR*^−^ mutant), in which the absence of the CPR blocked the production of astaxanthin, the production of this pigment was still possible in strain CBS.*Δdap1*, although its production decreased drastically to approximately 3% (Fig. 3 and Table 2). This observed phenomenon suggests that *DAP1* is necessary but not indispensable for the activity of CrtS, unlike the CrtR enzyme. Likewise, the deletion of *DAP1* in *X. dendrorhous* affected the production of sterols. The CBS.*Δdap1* mutant was more sensitive than the wild-type strain to azole drugs, suggesting that sterol biosynthesis was also affected in this mutant. Ergosterol, the main sterol produced by the yeast *X. dendrorhous*, was drastically reduced in the *DAP1* deletion mutant, and the yeast accumulated two other metabolites with a sterol spectrum: peak 1_(12min)_ and peak 2_(17.2min)_, whose retention times coincide with potential sterols that accumulated in strain CBSTr. In this last strain, the proportion of ergosterol decreased, but this decrease was not as pronounced as that in strain CBS.*Δdap1*. Interestingly, although the P450 electron donor systems CYB5/CBR and CrtR in strain CBS.*Δdap1* should have been functional, this was not enough to maintain wild-type ergosterol levels. Also, the CBSTr strain that does not have the CPR CrtR, is unable to produce astaxanthin (17), so the P450 enzyme (CrtS) involved in this process needs CrtR as an electron donor. Although it is possible that CrtR provides both electrons required for the activity of CrtS, it is also possible that an alternative electron donor, such as CYB5, donates the second electron, as described in other P450 reactions. Then, CrtS may only require CrtR to provide the first electron, and an alternative electron donor could donate the second one needed for the P450 cycle. Then, considering the structural similarity between Dap1 and CYB5, our results do not exclude the possibility that Dap1 may function as an electron donor providing the second electron involved in P450 reactions.

Heme binding is the only known biological function of Dap1. In the yeast *S. cerevisiae*, the exogenous addition of this compound (hemin) was sufficient to reverse the altered sterol composition in *Δdap1* mutants, a phenomenon that was analyzed by gas chromatography (11), suggesting that Dap1 is involved in the transport of heme and functions as a kind of chaperone for P450s. Considering this, the effect of the exogenous addition of this compound on the CBS.*Δdap1* mutant was analyzed. The addition of hemin reverted the growth defect in medium supplemented with clotrimazole but did not revert the altered color phenotype in mutant CBS.*Δdap1*. When the composition of sterols at the exponential phase of growth when hemin was added was analyzed, new unidentified metabolites appeared in the chromatogram; these metabolites are probably sterols, as their spectra were similar to that of sterols, which could mediate resistance to clotrimazole in this strain. Although no phenotypic differences in the pigmentation of the strain were observed when hemin was added, a slight increase in intermediary carotenoids between β-carotene and astaxanthin was detected. These last results may be because hemin was added at the exponential phase growth when the synthesis of carotenoids is induced and not at the stationary phase when these metabolites accumulate. Unfortunately, to the best of our knowledge, there is no such rapid and sensitive test to evaluate the effect of hemin on carotenogenesis as it is the addition of azoles in the culture medium to evaluate the effect of hemin on sterol biosynthesis. Then, considering our results, it is not possible to conclude that hemin did not affect carotenogenesis in the deletion mutant of *DAP1*.

Transcript levels of the P450-encoding genes *CYP61* and *CYP51* and the electron donor gene *crtR* were higher in the CBS.*Δdap1* strain. However, this increase in transcript level would not be sufficient to maintain wild-type ergosterol and astaxanthin production. Therefore, the deletion of *DAP1* in *X. dendrorhous* affected these biosynthetic pathways at another level of regulation. Given the variation in sterol composition in this strain, the increase in transcript levels of the mentioned genes could be due to activation of the transcription factor Sre1 (20, 41). To evaluate this possibility, strain CBS.*sre1*^*−*^*.Δdap1* was constructed, and as expected, the transcript levels of potential Sre1 targets were decreased to levels similar to those in strain CBS.*sre1*^*−*^. However, strain CBS.*sre1*^*−*^ has a wild-type color phenotype, but strain CBS.*sre1*^*−*^*Δdap1* was more orange than the wild-type strain and paler than the single CBS.*Δdap1* mutant strain. Then, the *sre1*^−^ mutation intensified the CBS.*Δdap1* mutant phenotype, and in addition to the absence of Dap1, which positively regulates the P450s involved in sterol or carotenoid biosynthesis, the transcript levels of Sre1 gene targets involved in the synthesis of isoprenoids were also reduced. These results also support the hypothesis of post-transcriptional regulation of P450s by Dap1. In addition, the co-IP assay results supported the interaction of Dap1 with the P450s Cyp51 and Cyp61, as was reported for *S. pombe* (8), and with CrtS in *X. dendrorhous*, providing a new role for Dap1: the regulation of carotenogenesis.

Regulation of carotenogenesis by Dap1 in *X. dendrorhous* would occur specifically at the steps in which astaxanthin is synthesized from β-carotene by the regulation of CrtS at the protein level. In this regard, several attempts have been made to obtain astaxanthin-overproducing *X. dendrorhous* strains. For example, when the carotenogenic gene *crtYB* (encoding a phytoene-β-carotene synthase) was overexpressed, higher carotenoid levels were reached; however, this increase was mainly because of the higher β-carotene content but not that of astaxanthin (43). Similarly, overexpression of the *crtE* gene (which encodes a geranylgeranyl pyrophosphate that catalyzes an early step of carotenogenesis (44)) enhanced overall carotenoid synthesis, but the production of astaxanthin did not increase in the same way (45). These examples support that the steps in which astaxanthin is biosynthesized from β-carotene in *X. dendrorhous* have certain limitations. Based on the results of our current work, we speculate that this limitation is due to Dap1. Therefore, the overexpression of *DAP1* with other carotenogenic genes is a potential strategy to obtain astaxanthin-overproducing *X. dendrorhous* strains.

Finally, our results provide new insights into the mechanisms of isoprenoid biosynthesis regulation in *X. dendrorhous*, pointing to Dap1 as a potential target to enhance the production of carotenoids, specifically astaxanthin, in this yeast.

## Data availability

All data are contained within the article.

## Supplemental data

This article contains supplemental data.

## Conflict of interest

The authors declare that they have no conflicts of interest with the contents of this article.
