# Supplementary material for: Damage response protein 1 (Dap1) functions in the synthesis of carotenoids and sterols in Xanthophyllomyces dendrorhous
Source: J Lipid Res. 2022 Feb 2;63(3):100175. doi: 10.1016/j.jlr.2022.100175 (PMC8953664; doi:10.1016/j.jlr.2022.100175)
Supplement: Supplemental Figures S1–S5 and Table S1 [file mmc1.docx]

**SUPPLEMENTAL INFORMATION:**

**Damage response protein 1 (Dap1) functions in the synthesis of carotenoids and sterols in *Xanthophyllomyces dendrorhous***

Ana-María González^1^, Maximiliano Venegas^1^, Salvador Barahona^2^_,_ Melissa Gómez^1^, María-Soledad Gutiérrez^1^, Dionisia Sepúlveda^2^, Marcelo Baeza^1,2^, Víctor Cifuentes^1,2^, Jennifer Alcaíno^1,2^.

^1^Departamento de Ciencias Ecológicas, Facultad de Ciencias, Universidad de Chile, Santiago, Chile

^2^Centro de Biotecnología, Facultad de Ciencias, Universidad de Chile, Santiago, Chile

**
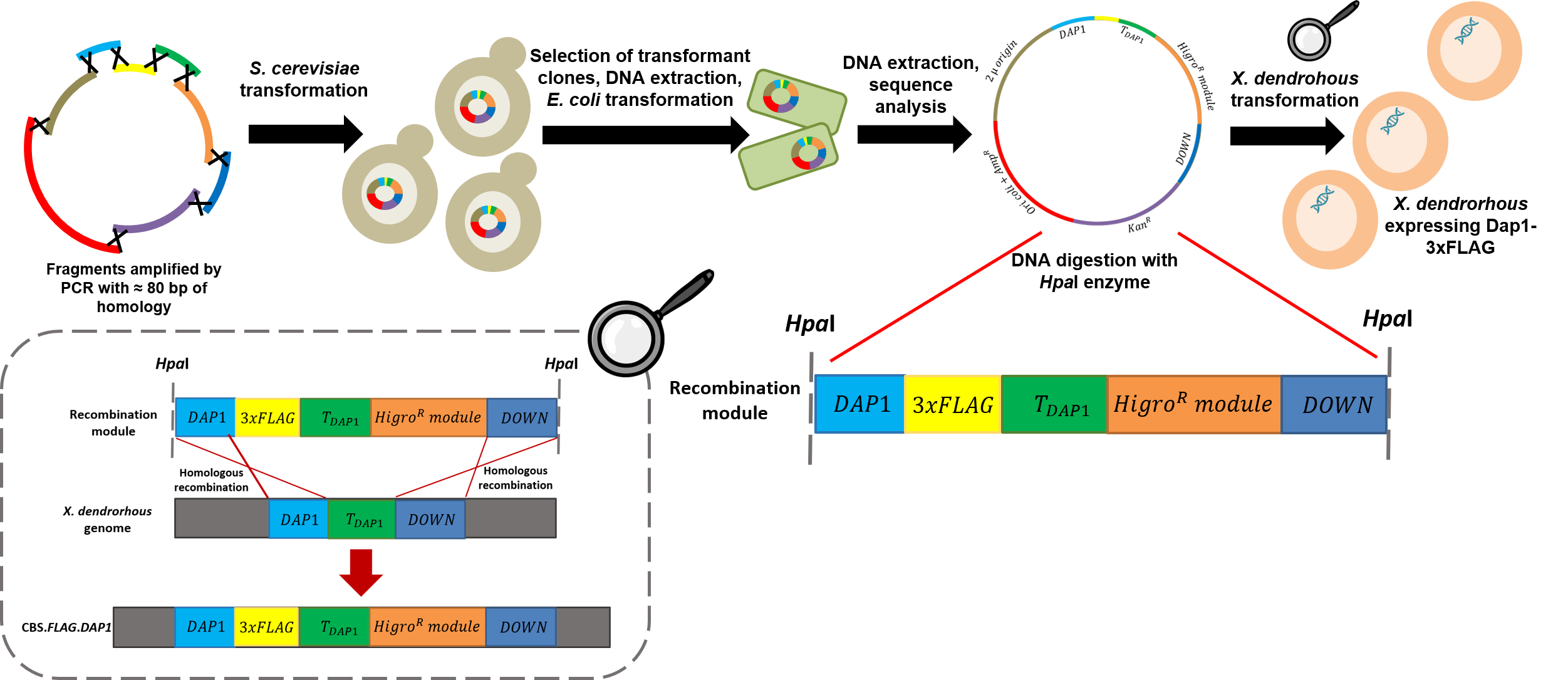
**

**Supplemental Figure S1. Plasmid construction by DNA assembler and homologous recombination in *X. dendrorhous*.** The construction of strain CBS.*FLAG*.*DAP1* is used as an example. Eight DNA fragments with complementary ends were PCR amplified and used to transform *S. cerevisiae* to allow their assembly *in vivo* through homologous recombination. Transformed *S. cerevisiae* clones were identified by their resistance to G-418. The total DNA of the positive clones was used to transform *E. coli* by electroporation. Positive *E. coli* clones were selected by ampicillin resistance, and the presence of the resistance module was verified by colony PCR. Then, plasmid DNA was recovered and digested with *Hpa*I to release the recombination module, which was used to transform *X. dendrorhous* by electroporation. The expected double homologous recombination event to generate the transforming strain is shown enclosed in a dotted line.


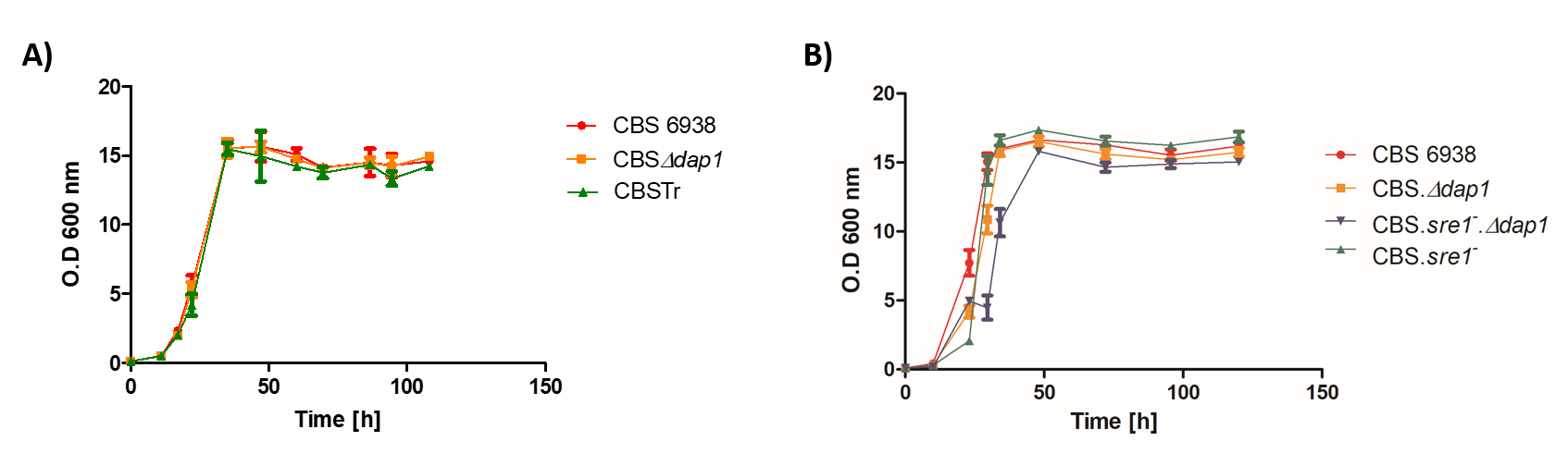


**Supplemental Figure S2. *X. dendrorhous* strain growth curves. Strains were cultured at 22°C with constant agitation in YM medium. A)** Growth curves of strains CBS 6938 (wild type), CBS.*Δdap1*, and CBSTr. **B)** Growth curves of strains CBS 6938 (wild type), CBS.*Δdap1*, CBS.*sre1^-^.Δdap1* and CBS.*sre1^-^*. After 120 h of culture, samples were taken for further analysis (carotenoid and sterol content determination, RNA and protein extraction, according to the corresponding analysis). Data are the mean ± standard deviation of three biological replicates.


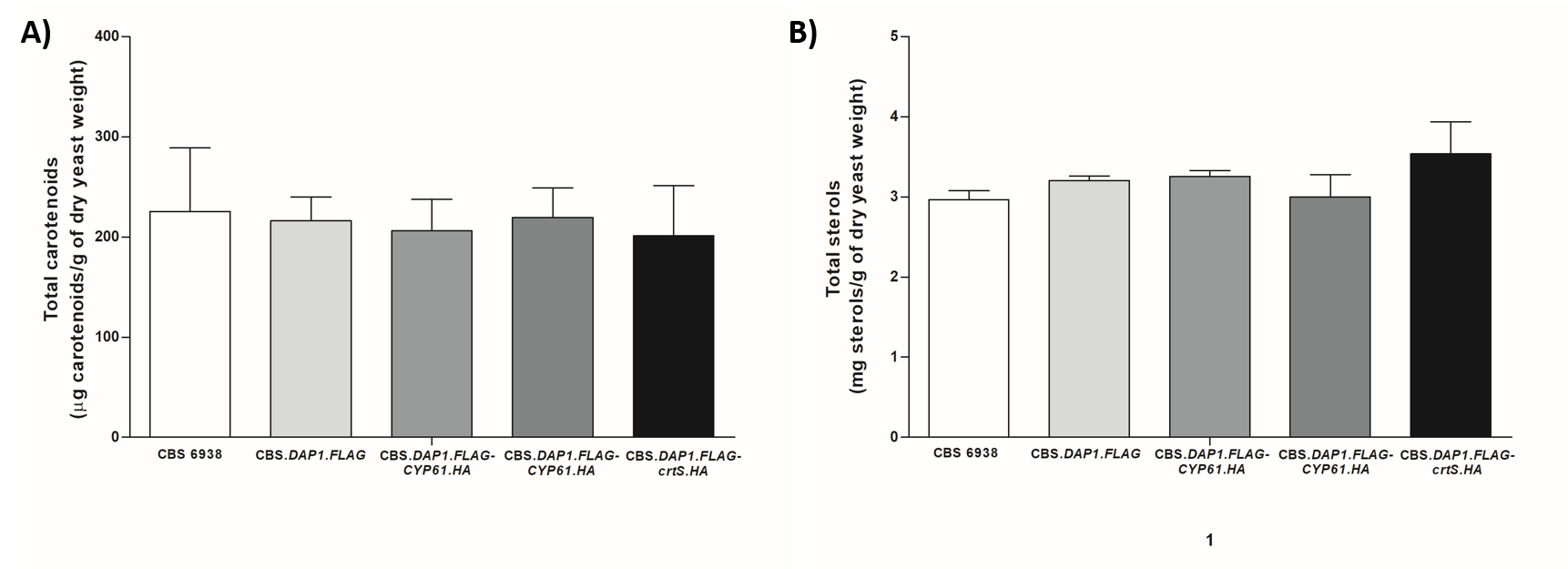
 **Supplemental Figure S3. Production of carotenoids and sterols in strains CBS 6938, CBS.*DAP1.FLAG,* CBS.*DAP1.FLAG-CYP61.HA,* CBS.*DAP1.FLAG-CYP51.HA* and CBS*.DAP1.FLAG-crtS.HA.* A)** Carotenoid and **B)** sterol production is expressed as μg of carotenoids or mg of sterols per g dry yeast, respectively. The mean ± standard deviation of three independent cultures of each strain is shown. One-way ANOVA followed by the Tukey post-test was used as a statistical test. No significant differences were observed among strains.

**
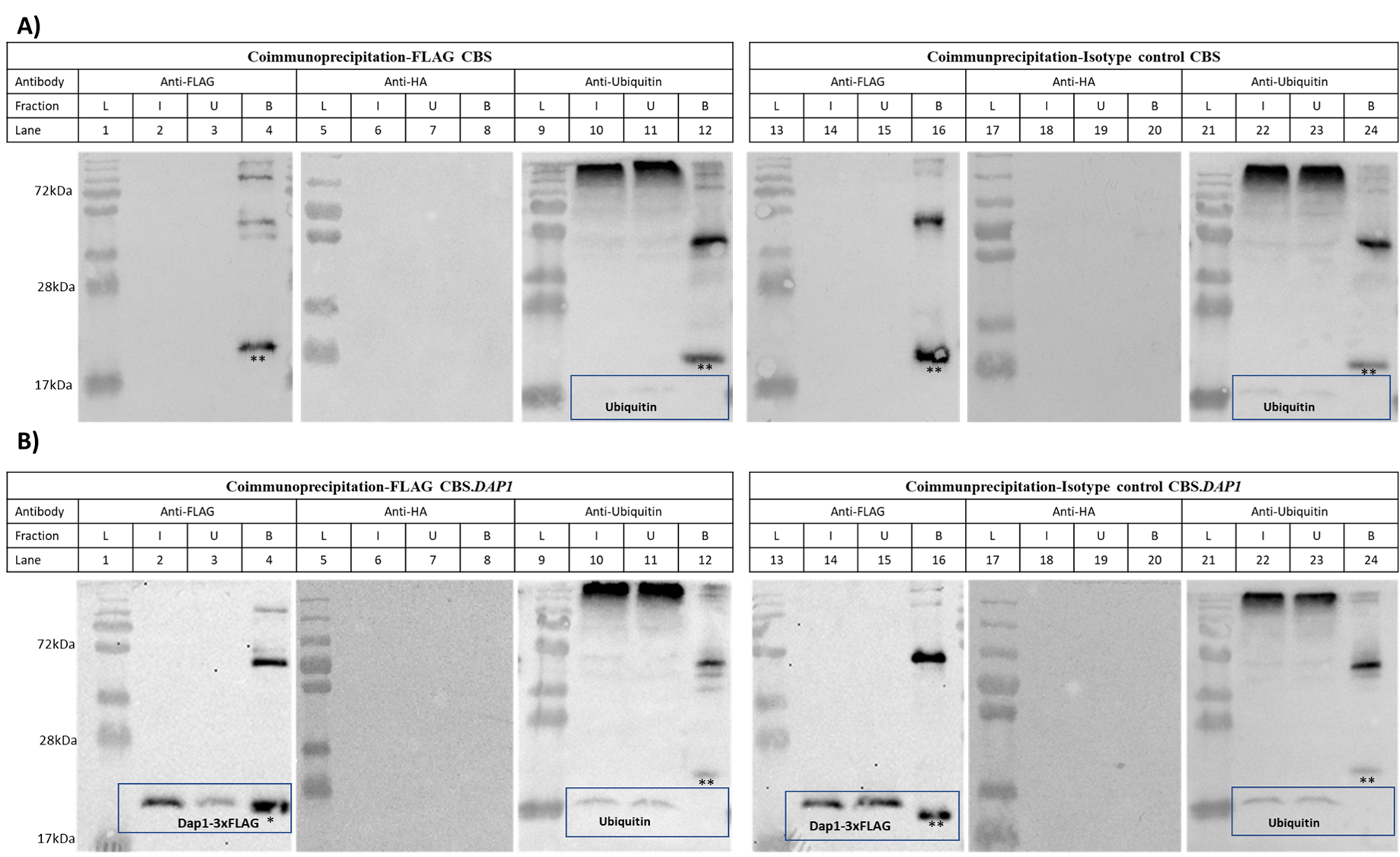
**

**Supplemental Figure S4. Control of coimmunoprecipitation assays.** Protein extracts from strains A) CBS 6938 and B) CBS.*DAP1.FLAG* were subjected to immunoprecipitation with anti-FLAG antibody or mouse IgG1 kappa monoclonal as an isotype control . The input (I), unbound (U), and bound (B) fractions were analyzed by western blotting with anti-FLAG (Dap1-3xFLAG immunoprecipitation control), anti-HA (to evaluate the coimmunoprecipitation with target proteins) or anti-ubiquitin (as a coimmunoprecipitation specificity control) antibodies. In western blotting with anti-FLAG and anti-ubiquitin, anti-mouse IgG H&L-Peroxidase was used as the secondary antibody (anti-HA fused to peroxidase). The expected size of the proteins was approximately 21.3 and 16.8 kDa for Dap1-3xFLAG and ubiquitin, respectively: the blue boxes frame the position of the analyzed bands in each gel. * Indicates a double band [one corresponding to the Dap1-3xFLAG protein (higher band) and the other to the light chain of the antibody used for immunoprecipitation (lower band)] and ** indicates a single band corresponding to the light chain of the antibody used for immunoprecipitation. PageRuler Plus 10-250 kDa was used as a molecular weight standard.

**
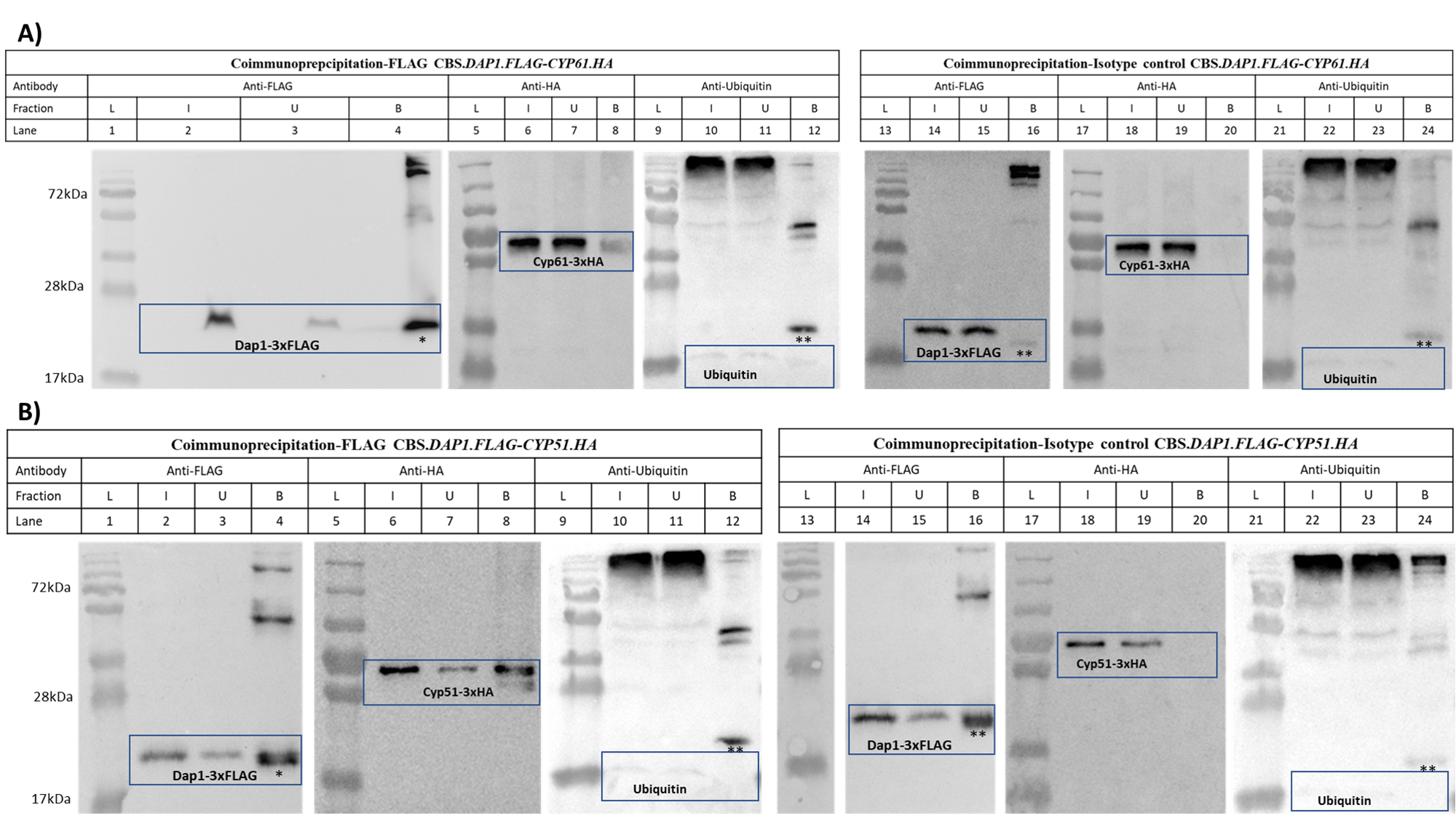
**

**Supplemental Figure S5. Coimmunoprecipitation of Dap1 with Cyp61 and Cyp51.** Protein extracts from strains A) CBS.*DAP1.FLAG-CYP61.HA* and B) CBS.*DAP1.FLAG-CYP51.HA* strain were subjected to immunoprecipitation with anti-FLAG antibody or mouse IgG1 kappa monoclonal as an isotype control . The input (I), unbound (U), and bound (B) fractions were analyzed by western blotting with anti-FLAG (Dap1-3xFLAG immunoprecipitation control), anti-HA (to evaluate the coimmunoprecipitation of Cyp61-3xHA and Cyp51-3xHA with Dap1-3xFLAG) or anti-ubiquitin (as a coimmunoprecipitation specificity control) antibodies. In western blotting with anti-FLAG and anti-ubiquitin, anti-mouse IgG H&L-Peroxidase was used as the secondary antibody (anti-HA fused to peroxidase). Lane 13 in panel B is the same as lane 13 from Supplemental Figure S4 A as both sets of experiments were loaded in the same gel. The expected size of the proteins was approximately 21.3; 63.1, 65.2 and 16.8 kDa for Dap1-3xFLAG, Cyp61-3xHA, Cyp51-3xHA and ubiquitin, respectively: the blue boxes frame the position of the analyzed bands in each gel. * Indicates a double band [one corresponding to the Dap1-3xFLAG protein (higher band) and the other to the light chain of the antibody used for immunoprecipitation (lower band)] and ** indicates a single band corresponding to the light chain of the antibody used for immunoprecipitation. PageRuler Plus 10-250 kDa was used as a molecular weight standard.

**Supplemental Table S1. Primers used in this work.**

| **Primer name** | **Sequence (5’-3’)** | **Target and/or special feature** |
| --- | --- | --- |
| ***DAP1* gene deletion** | | |
| CTR_F | TGGCCTACTCGATCGTATTTC | *DAP1* gene |
| CTR_R | GACATTTCAGAAGGCGTGAGG | *DAP1* gene |
| DAP1_ATG.F | ATGTCACTCTCGATCCTTGTC | *DAP1* gene |
| DAP1_STOPR2.R | TCATGAATCGGTGTTCTCTAT | *DAP1* gene |
| gDel_DAP1_HpaI.F | ACCACCCTGACGTTAACACTACTTTTAGCTCTCCTTTC | *DAP1* gene promoter |
| gDel_DAP1.F | CGACCGTAGGAAAGAAAGAGA | *DAP1* gene promoter |
| Pre_del_DAP1.F | TTAGAGGCGAGGTCTTTGTTC | *DAP1* gene promoter |
| gDel_DAP1.R | CCTTTCCGATCTGTACTCTCA | *DAP1* gene terminator |
| DAP1_int_DOWN_R | GCGGGTATACGATCAGCTTTA | *DAP1* gene terminator |
| gDel_DAP1_HpaI.R | CTAAAAGTAGTGTTAACGTCAGGGTGGTGAACGGATT | *DAP1* gene terminator |
| Pre_del_DAP1.R | TCTTCCTTCTCTACTGACCTG | *DAP1* gene terminator |
| ***CYP61* gene** | | |
| CYP61.F | CTGAGCCCTGTCTTGTTGCC | *CYP61* gene |
| CYP61Ff.Fw | TCGAGTACGCCTACATGCAC | *CYP61* gene |
| Cyp61-cF2.Rv | CGATCTCCGCGATATAGGAT | *CYP61* gene |
| CYP61_int_UP.F | TCGTTACCTAAGAGACGAG | *CYP61* gene promoter |
| Cyp61dw.Rv | GTAACTGGAGCGAGCCAAAG | *CYP61* gene terminator |
| CYP61_int_DOWN_R | AACCGATCGAAGAAGATGCAC | *CYP61* gene terminator |
| ***CYP51* gene** | | |
| CYP51.F | CAAGGATGTCGTATACGATTGC | *CYP51* gene |
| CYP51.R | GAGGTGTGCTGACCGGCC | *CYP51* gene |
| CYP51Ff.Fw | CGGGAGAGCAGTACGAGAAC | *CYP51* gene |
| CYP51.Re.R | CTTCCTGCAGACAAGACACG | *CYP51* gene promoter |
| CYP51_int_UP.F | TGACACGTAGCAGGATTCTTG | *CYP51* gene promoter |
| cCYP51.R | TCGCCCAAATTTGAAGAGAC | *CYP51* gene terminator |
| CYP51_int_DOWN.R | CGAACAGTATCCTGACAGGC | *CYP51* terminator |
| ***crtS* gene** | | |
| CrtS.bF | CTCGAGTGCTCAGAATTGCTAC | *crtS* gene |
| CrtS-cF | TGGATGTTTCACCGACTCTC | *crtS* gene |
| RTCrtS.Rv | CGTCGGGAAACTGCTAATCCTTGA | *crtS* gene |
| cCrtS.Fw | GGTTGTCCCATGTGCTTCTT | *crtS* gene promoter |
| cCrtS.Rv | CAGAAGCATGAAGAGGCTGA | *crtS* gene terminator |
| CrtSDOWN2.Rv | TGCTGTTCATCGTTAGTCG | *crtS* gene terminator |
| CrtS_int_DOWN.Rv | ATCGGCTTCGAGAATTCCG | *crtS* gene terminator |
| **hygromycin B and Zeocin module** | | |
| P.Tef.F | ATCGGCTCATCAGCCGACAGT | *EF-1α* promoter |
| T.gpd.R | ATGAGAGATGACGGAGATGAT | *gpd* terminator |
| H-out.R | TCCATCACAGTTTGC | Hygromycin module |
| H-out2.F | CTCGCCGATAGTGGAAACCGAC | Hygromycin module |
| Zeo_int_F | GAGTGGTCGGAGGTCGTGTC | Zeocin module |
| Zeo_int_R | CGTGACCCTGTTCATCAGC | Zeocin module |
| ***DNA assembler*** | | |
| A.Fw | TATCATGCGTCAATCGTATGTGATGCTGGTCGCTATACTGGCATTAAGCGCGGCGGGTG | pBS |
| A.Rv | AGTCCGTGGAATTAATTCTCATCTTTGACAGCTTATCATCGATAAGTGCGCGGAACCCCT | pBS |
| H2.Fw | GCCGTGTTTGAATGCAAGTTTGCTCGCTGTTGATCGTTCGTTAACGACATGGAGGCCCAG | pFA6a |
| H.Rv | GCGGTCACGCTGCGCGTAACCACCACACCCGCCGCGCTTAATGCCAGTATAGCGACCAGC | pFA6a |
| up.Fw | TTTTGAGCAATGTTTGTGGAAGCGGTATTCGCAATGGTTAACCGGTGCTTGCATAAGGCA | *DAP1* gene |
| dap1FLAG.Rv | ATATCATGATCTTTATAATCACCGTCATGGTCTTTGTAGTCTGAATCGGTGTTCTCTATC | *DAP1* gene |
| FLAG.Fw | GTATATCATCTGTGGGTCATTGATAGAGAACACCGATTCAGACTACAAAGACCATGACGG | pFlagTEM1 |
| FLAG.Rv | GACGAGAAAACAAATACCTTGCTGATCGAAAGGAGAGCTCATTTATCGTCGTCATCTTTG | pFlagTEM1 |
| Tdap1.Fw | AAGATCATGATATCGACTACAAAGATGACGACGATAAATGAGCTCTCCTTTCGATCAGCA | *DAP1* gene terminator |
| Tdap1.Rv | AAGAGCTTGTGTCGGATGAACTGTCGGCTGATGAGCCGATTCCTCAATTTACAATGGCCG | *DAP1* gene terminator |
| mResist.Fw | ATCCCCATGAATGACGCACCCGTATCGGCCATTGTAAATTGAGGAATCGGCTCATCAGCC | Hygromycin B module |
| mResist.Rv | GAAAACAAATACCTTGCTGATCGAAAGGAGAGCTAAAAGTAGTATCATGAGAGATGACGG | Hygromycin B module |
| down.Fw | ACATCTGTTGACCATCACCATCATCTCCGTCATCTCTCATGATACTACTTTTAGCTCTCC | *DAP1* gene terminator |
| down.Rv | CAAGACTGTCAAGGAGGGTATTCTGGGCCTCCATGTCGTTAACGAACGATCAACAGCGAG | *DAP1* gene terminator |
| B.Fw | GAATGTATTTAGAAAAATAAACAAATAGGGGTTCCGCGCACTTATCGATGATAAGCTGTC | pYES2 |
| B.2 Rv | GGGGAAGGTGTTTGCACTGCCTTATGCAAGCACCGGTTAACCATTGCGAATACCGCTTCC | pYES2 |
| B2CYP61.Rv | AAAAGAGTGCGTTAATAGGCCACAAACCTGATTCCGTTAACCATTGCGAATACCGCTTCC | pYES2 |
| UPCYP61.Fw | TTTTGAGCAATGTTTGTGGAAGCGGTATTCGCAATGGTTAACGGAATCAGGTTTGTGGCC | *CYP61* gene |
| assCYP61.Rv | CATAGGGATAGCCAGCGTAATCTGGAACATCGTATGGGTAGAAGGAAGGTTTAGGTCGAG | *CYP61* gene |
| 3xHACYP61.Fw | TGGTCTTCACCTCAAGTTTACCCCTCGACCTAAACCTTCCTTCTACCCATACGATGTTCC | 3xHA sequence |
| 3xHACYP61.Rv | AGGTATGTATAGAGAGATGATAAGCCAGATAGGTGCTCATTTCAAGCGTAATCTGGAACG | 3xHA sequence |
| tCYP61.Fw2 | ATGCAGGATCCTATCCATATGACGTTCCAGATTACG CTTGAAATGAGCACCTATCTGGCT | *CYP61* gene terminator |
| tCYP61.Rv | AAGAGCTTGTGTCGGATGAACTGTCGGCTGATGAGCCGATGTAACGGCGTTTCCTTCTGC | *CYP61* gene terminator |
| mresistCYP61.Fw | TTCAATGCAGAACCAGAGTAACAATGCAGAAGGAAACGCCGTTACATCGGCTCATCAGCC | Zeocin module |
| mresistCYP61.Rv | GAGGTATGTATAGAGAGATGATAAGCCAGATAGGTGCTCATTCATCATGAGAGATGACGG | Zeocin module |
| DownCYP61.Fw | ATCTGTTGACCATCACCATCATCTCCGTCATCTCTCATGATGAATGAGCACCTATCTGGC | *CYP61* gene terminator |
| DownCYP61.Rv | AGACTGTCAAGGAGGGTATTCTGGGCCTCCATGTCGTTAACGACTCTGCTAAGGGAGATG | *CYP61* gene terminator |
| H2CYP61.Fw | GATGAGTCCTTTTTCTTTCTCATCTCCCTTAGCAGAGTCGTTAACGACATGGAGGCCCAG | pFA6a |
| B2CYP51.Rv | CAAAGGCGAAGAGGAAGAGAGAGTCCGGTATTGGGGTTAACCATTGCGAATACCGCTTCC | pYES2 |
| UPCYP51.Fw | ACTTTTGAGCAATGTTTGTGGAAGCGGTATTCGCAATGGTTAACCCCAATACCGGACTCT | *CYP51* gene |
| assCYP51.Rv | CATAGGGATAGCCAGCGTAATCTGGAACATCGTATGGGTACGCGGCAGCCTTTCGAGGCT | *CYP51* gene |
| 3xHACYP51.Fw | TACCATCCACTACAGGAAGCGACAGCCTCGAAAGGCTGCCGCGTACCCATACGATGTTCC | 3xHA sequence |
| 3xHACYP51.Rv | AGAGAGGAGAAGAAGAAACTTCTCAAGGAAAAGCGTCTGATTCAAGCGTAATCTGGAACG | 3xHA sequence |
| TCYP51.Fw2 | TGCAGGATCCTATCCATATGACGTTCCAGATTACGCTTGAATCAGACGCTTTTCCTTGAG | *CYP51* gene terminator |
| tCYP51.Rv | AAGAGCTTGTGTCGGATGAACTGTCGGCTGATGAGCCGATTGTCTTCTAGCCAAACCCCG | *CYP51* gene terminator |
| mResistCYP51.Fw | AGTCGATAGATGCTGGGCAGAGAAGCGGGGTTTGGCTAGAAGACAATCGGCTCATCAGCC | Zeocin module |
| mResistCYP51.Rv | CTTTTCTTGCTTGTCTCACTCTGGAAATTTCATCATCGTCTCCATCATGAGAGATGACGG | Zeocin module |
| DownCYP51.Fw | GGAGACGATGATGAAATTTCCCTGTTGACCATCACC ATCATCTCCGTCATCTCTCATGAT | *CYP51* gene terminator |
| DownCYP51.Rv | GACTGTCAAGGAGGGTATTCTGGGCCTCCATGTCGTTAACTAGAAGTTATGGATCCCGCG | *CYP51* gene terminator |
| H2CYP51.Fw | TAGAGATCCTATTCTCTTGCGCGGGATCCATAACTTCTAGTTAACGACATGGAGGCCCAG | pFA6a |
| B2CrtS.Rv | TTCGGCAAGGACCGATCGAGCCAGATGTCGCTCTAGGGCCCCATTGCGAATACCGCTTCC | pYES2 |
| UPCrtS.Fw | TTTTGAGCAATGTTTGTGGAAGCGGTATTCGCAATGGGGCCCTAGAGCGACATCTGGCTC | *crtS* gene |
| assCrtS.Rv | GTCATAGGGATAGCCAGCGTAATCTGGAACATCGTATGGGTATTCGACCGGCTTGACCTG | *crtS* gene |
| 3XHACrtS.Fw | GAAGGAGGGGTACCAGATGCGTTTGCAGGTCAAGCCGGTCGAATACCCATACGATGTTCC | 3xHA sequence |
| 3XHACrtS.Rv | AGAAACAAATCGATACAAAGAAGGCATTGTCCTAGTCACACTCAAGCGTAATCTGGAACG | 3xHA sequence |
| tCrtS.Fw2 | ATGCAGGATCCTATCCATATGACGTTCCAGATTACGCTTGAGTGTGACTAGGACAATGCC | *crtS* gene terminator |
| tCrtS.Rv | AAGAGCTTGTGTCGGATGAACTGTCGGCTGATGAGCCGATAGTTGTCTGGATGGAGTTCC | *crtS* gene terminator |
| mResistCrtS.Fw | TGACCGAAGTTTCTCCACAATCCGGGGAACTCCATCCAGACAACTATCGGCTCATCAGCC | Zeocin module |
| mResistCrtS.Rv | AGAGAGGAAAGTGAGGTCGATTAGATCGACATGGTGAGAGAATCATGAGAGATGACGGAG | Zeocin module |
| DownCrtS.Fw | CATCTGTTGACCATCACCATCATCTCCGTCATCTCTCATGATTCTCTCACCATGTCGATC | *crtS* gene terminator |
| DownCrtS.Rv | CAAGACTGTCAAGGAGGGTATTCTGGGCCTCCATGTCGGGCCCTCGTCTTAGGGAATCGG | *crtS* gene terminator |
| H2CrtS.Fw | CACCTCCACTAACGAACACCGACCGATTCCCTAAGACGAGGGCCCGACATGGAGGCCCAG | pFA6a |
| **RT-qPCR** | | |
| RT_CYP61.F | CATGGAAAGGTTCACGCCGAGTAT | *CYP61* gene |
| RT_CYP61.R | AAAGACCCGGAGGGAAGTTTCCAT | *CYP61* gene |
| RT-CYP51.F | CAGCTCGCTCAGTTGATTCCTAGA | *CYP51* gene |
| RT-CYP51.R | ATGTGAACAGATCGCCGTGCTT | *CYP51* gene |
| crtS_RT.F | TATCTCTGGACCCAGAGCTTGC | *crtS* gene |
| crtS_RT.R | ACCTGCAAACGCATCTGGTAC | *crtS* gene |
| mcrtRF-RT | CTGGGAAACAAGACC | *crtR* gene |
| mcrtRF-RT | CGGAACCTCGGTTACG | *crtR* gene |
| hmgS.RT2.F | AAGCAGGTTGAGCCTGGAATGA | *HMGS* gene |
| hmgS.RT2.R | AAAGCGGTTGAGCTCTTGACCT | *HMGS* gene |
| mActF-RT | CCGCCCTCGTGATTGATAAC | *ACT* gene |
| mActR-RT | TCACCAACGTAGGAGTCCTT | *ACT* gene |
